# Gene silencing, knockout and over-expression of a transcription factor *ABORTED MICROSPORES (SlAMS)* strongly affects pollen viability in tomato (*Solanum lycopersicum*)

**DOI:** 10.1186/s12864-022-08549-x

**Published:** 2022-05-05

**Authors:** Huihui Bao, Yumei Ding, Fei Yang, Jie Zhang, Junjun Xie, Chongyan Zhao, Kanghua Du, Yawen Zeng, Kai Zhao, Zuosen Li, Zhengan Yang

**Affiliations:** 1 College of Horticulture and Landscape, Yunnan Agricultural University, Kunming, Yunnan 650201 People’s Republic of China; 2 Biotechnology and Germplasm Resources Institute, Yunnan Academy of Agriculture Sciences, Kunming, Yunnan 650205 People’s Republic of China; 3 College of Food Science and Technology, Yunnan Agricultural University, Kunming, Yunnan 650201 People’s Republic of China

**Keywords:** Tomato, *SlAMS*, VIGS, Gene knockout, Over-expression

## Abstract

**Background:**

The tomato (*Solanum lycopersicum* L.) is an economically valuable crop grown worldwide. Because the use of sterile males reduces the cost of F1 seed production, the innovation of male sterility is of great significance for tomato breeding. The *ABORTED MICROSPORES *gene* (AMS),* which encodes for a basic helix-loop-helix (bHLH) transcription factor, has been previously indicated as an essential gene for tapetum development in *Arabidopsis* and rice. To determine the function of the *SlAMS* gene (*AMS* gene from *S. lycopersicum*) and verify whether it is a potential candidate gene for generating the male sterility in tomato, we used virus-induced gene silencing (VIGS), CRISPR/Cas9-mediated genome editing and over-expression technology to transform tomato via *Agrobacterium* infection.

**Results:**

Here, the full-length *SlAMS* gene with 1806 bp from *S. lycopersicum* (Accession No. MK591950.1) was cloned from pollen cDNA. The results of pollen grains staining showed that, the non-viable pollen proportions of *SlAMS*-silenced (75%), −knockouted (89%) and -overexpressed plants (60%) were significantly higher than the wild type plants (less than 10%; *P* < 0.01). In three cases, the morphology of non-viable pollen grains appeared tetragonal, circular, atrophic, shriveled, or otherwise abnormally shaped, while those of wild type appeared oval and plump. Furthermore, the qRT-PCR analysis indicated that *SlAMS* in anthers of *SlAMS*-silenced and -knockouted plants had remarkably lower expression than in that of wild type (*P* < 0.01), and yet it had higher expression in *SlAMS-*overexpressed plants (*P* < 0.01).

**Conclusion:**

In this paper, Our research suggested alternative approaches to generating male sterility in tomato, among which CRISPR/Cas9-mediated editing of *SlAMS* implied the best performance. We also demonstrated that the downregulation and upregulation of *SlAMS* both affected the pollen formation and notably led to reduction of pollen viability, suggesting *SlAMS* might be essential for regulating pollen development in tomato. These findings may facilitate studies on clarifying the *SlAMS-*associated molecular regulatory mechanism of pollen development in tomato.

**Supplementary Information:**

The online version contains supplementary material available at 10.1186/s12864-022-08549-x.

## Introduction

The tomato (*Solanum lycopersicum* Mill.) is a valuable self-pollinating fruit crop that is cultivated globally [1]. The tomato is imminently suitable for genetic engineering and as a model plant due to its small genome [2, 3]. As the use of sterile males may reduce the cost of F_1_ hybrid seed production, male sterility has the widespread application value and great prospects in tomato breeding [4]. Thus, many researchers have attempted to develop and identify male-sterile tomato lines. At present, hybrid tomato seed production is dominated by artificial emasculation and pollination, which is time-consuming and labor-intensive. In addition, in existing male-sterile tomato lines, it is difficult to maintain pollen infertility and stamen degeneration to ensure complete sterility [5, 6]. As functional male sterility in tomato is also greatly affected by environmental conditions, these sterile types are rarely used for the production of tomato hybrids. Previously, various genes associated with male sterility in tomatoes have been mapped to various chromosomes using morphological and molecular markers. Based on studies of the molecular mechanisms regulating anther and pollen development in *Arabidopsis* and rice, abortive-pollen male-sterile tomato mutants have been artificially produced by knocking out key regulating genes [7].

Tapetum is the inner layer of plant anther wall. During the growth and development of plant pollen, the programmed cell death of tapetum provides necessary nutrients for the development of microspores [8]. Delayed or early degradation of the tapetum may adversely affect pollen development, leading to male sterility. Molecular techniques have previously been used to discover and clone genes related to tapetum development in rice [9], *Arabidopsis* [10] and petunias [11]. In *Arabidopsis,* the genes and transcription factors regulating early tapetum development include *SPOROCYTELESS* (*SPL*)*/NOZZLE* (*NZZ*), *AGAMOUS* (*AG*), *TAPETALDETERMINANT1* (*TPD1*), *EXTRA SPOROGENOUS CELLS/EXCESS MICROSPOREOCYTESL* (*EXS/EMS1*), *MITOGEN-ACTIVATED PROTEIN KINASES3/6* (*MPK3/MPK6*), *BARELY ANY MERISTEM1/2* (*BAM1/BAM2*), *ERECTA/ERECTA-LIKE1/2* (*ER/ERL1/ERL*2) [12–14], and the gene expressed in the later tapetum development were reported as *TAPETAL DEVELOPMENT AND FUNCTION1* (*TDF1*), *ABORTED MICROSPORES* (*AMS*), *MYELOBLASTOSIS103* (*MYB103*), and *MALE STERILITY1* (*MS1*) [15, 16]. For example, mutations of *SPL/NZZ* led to development failures in the tapetum, as well as the pollen sac and spore mother cell primordium [17]. The transcription factors, *DYSFUNCTIONAL TAPETUM1* (*DYT1*), *TDF1*, *AMS*, *MS188*, and *MS1* form a pathway that controls tapetum development [18, 19]. For example, *DYT1*, the most upstream regulator of tapetum development, affects many downstream genes associated with tapetum development and pollen wall formation by directly regulating *TDF1* [20].

The *AMS* gene encodes a basic helix-loop-helix-like (bHLH) transcription factor. It has been reported that *AMS* and its direct downstream regulator *MS188* are essential for tapetum development: *AMS* mutants developed abnormally enlarged tapetum cells and abortive microspores in *Arabidopsis* [21, 22]. In addition, the AMS protein combines with MS188 (or other MYB proteins) and bHLH010 (or other bHLH proteins) to regulate the expression of the sporopollenin synthesis gene [23]. *AMS* regulates the temporal and spatial expression of 23 genes, such as *CYTOCHROME 703A2* (*CYP703A2*), *POLYKETIDE SYNTHASE A/B* (*PKSA/PKSB*), *TETRAKETIDE-a-PYRONE REDUCTASE1* (*TKPR1*) and *CYP704B1*, precisely controlling several important life processes associated with pollen wall formation, including enamel and primary wall synthesis, as well as sporopollenin synthesis, transport, and deposition. *AMS* also affects microspore development, participates in the control of anther cell differentiation, and plays a role in biphasic regulation of tapetum development and pollen abortion in *Arabidopsis* [24, 25].

The *AMS* gene has been identified and characterized in several plants besides *Arabidopsis.* For example, in *Capsicum annuum* L., *CaAMS* was preferentially expressed in the tapetum during the tetrad and early-intermediate mononuclear stages; *CaAMS* downregulation led to the partial shortening of the capsicum flower, atrophy, unbroken stamens, and aborted pollen. By contrast, in *CaAMS*-silenced anthers, several genes involved in the formation of the outer pollen walls were downregulated [26]. Similarly, rice *TAPETUM DEGENERATION RETARDATION* (*TDR*), which was shown to be orthologous to *Arabidopsis AMS*, is preferentially expressed in the tapetum and encodes a putative bHLH protein; in rice, *TDR* is a key component gene of the molecular network regulating tapetum development and degeneration [16, 27]. Finally, it was shown that the silencing of the *AMS* homolog in melon (*Cucumis melo* L.) led to male sterility [4]. Thus, *AMS* may represent a candidate gene for the control of male sterility.

In this study, we aimed to determine whether *SlAMS* was a potential candidate gene for the development of male-sterile tomato lines*.* First*,* we cloned the tomato *SlAMS* gene using reverse transcription-polymerase chain reactions (RT-PCR), and predicted the physicochemical properties and structural characteristics of the AMS protein. Second, we verified *SlAMS* function using virus induced gene silencing (VIGS). Finally, we constructed an *SlAMS* knockout vector using the CRISPR/Cas9-mediated genome editing system, and an *SlAMS* overexpression vector using plasmid pCAMBIA2301. We then assessed the viability and morphology of the pollen grains produced by untransformed plants carrying either the *SlAMS* silencing, knockout, or overexpression vector. Our research might not only create the new male sterility materials which would be able to be used in tomato breeding, but also be significant for the further deeply understanding the mechanism of pollen development and regulation in tomato.

## Results

### Characterization of the tomato *AMS* gene

**Fig. 1:**
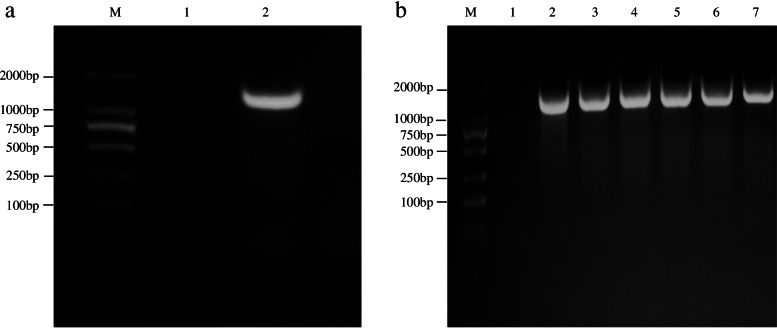
Electrophoresis gels image showing (**a**) RT-PCR cloning of the tomato *Aborted Microspores* (*SlAMS*) gene and (**b**) recombinant colony detection. Lane M: DL-2000 DNA marker; Lane 1: negative control; Lane 2: PCR products of *SlAMS* gene; Lanes 3–7: colonies

The tomato *SlAMS* gene was successfully cloned. The amplified fragment was about 1.8 kb long (Fig. 1a); six single colonies were verified using PCR (Fig. 1b). The full length of the tomato *SlAMS* gene was 1806 bp, which contained the complete open reading frame (ORF) region (Additional file 1). The cloned *SlAMS* sequence was submitted to GenBank (Accession No. MK591950.1). Nucleotide similarity analysis of *SlAMS* gene sequences indicated that the identity percentage of the cloned *SlAMS* gene varied from 78 to 100% when compared with the known sequences (Additional file 12), and the Solanaceae crops shared the higher sequence similarity (85% ~ 100%) as well.

**Fig. 2:**
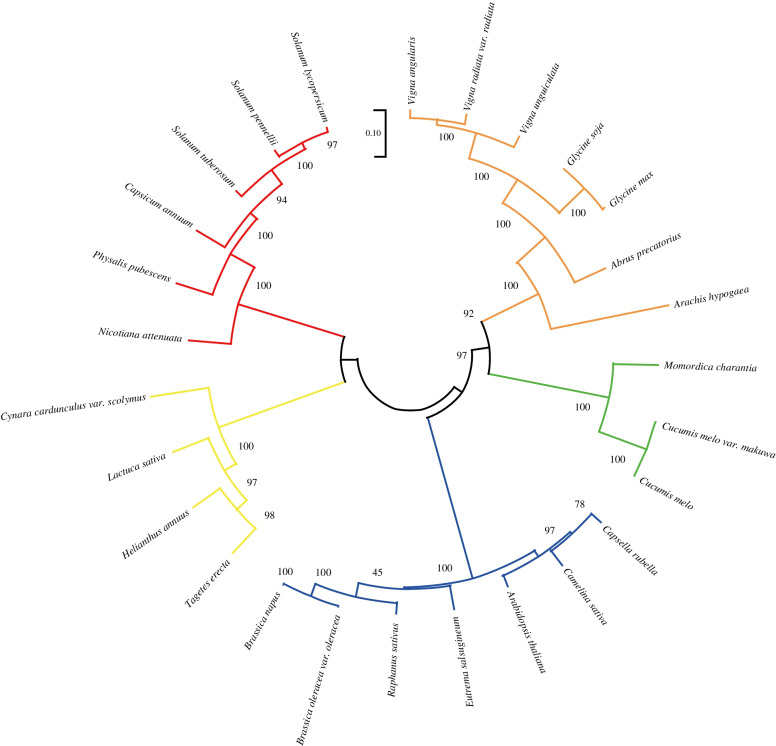
Phylogenetic tree among Aborted Microspores (AMS) proteins from various plant species. Plant families are shown in different colors: Solanaceae (red); Compositae (yellow); Cruciferae (blue); Cucurbitaceae (green); and Leguminosae (orange). Scale bar = 0.10 substitution rate

The phylogenetic analysis showed that the tomato SlAMS protein (GenBank ID: QDO73362.1) formed a larger, well-supported Solanaceae clade that included *S. pennellii* (XP_027774838.1), *S. tuberosum* (XP_0063515 93.1), *Capsicum annuum* (XP_016537577.1), *hysalis pubescens* (AZB50351.1) and *Nicotiana attenuata* (OIT32810.1; Fig. 2). The phylogenetic tree also identified genetic evolution relationship of AMS proteins in other economically important crops, such as *C. annuum*, *Helianthus annuus* (XP_022028120.1), *Brassica napus* (XP_022562176.1), *Raphanus sativus* (XP_018437312.1), and *Glycine max* (XP_025979976.1). Our phylogenetic tree indicated a close evolutionary relationship among the tomato SlAMS protein and other Solanaceae plants, suggesting that it may be generally conserved within families, and may have similar functions across the Solanaceae species.

**Fig. 3:**
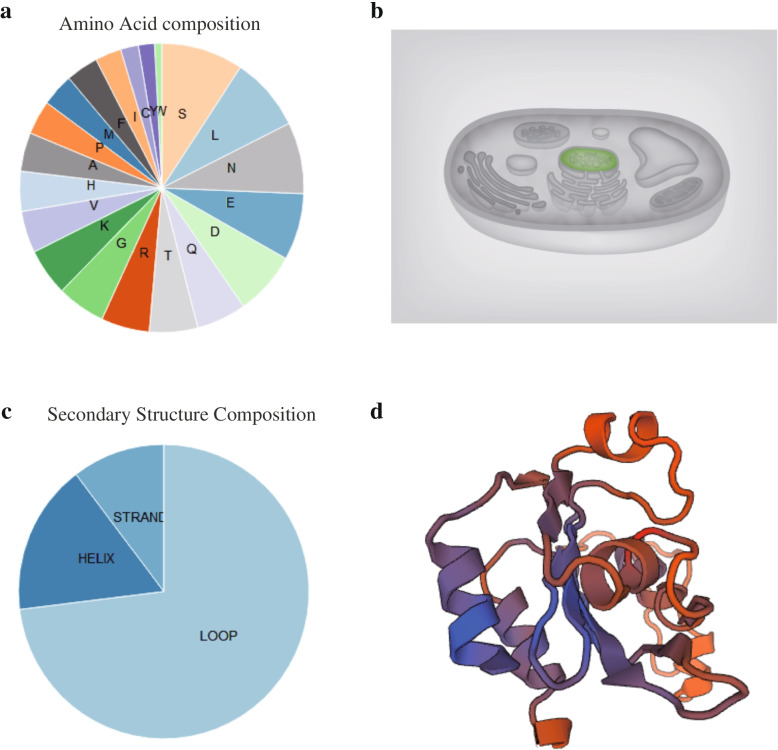
Characterization of the tomato Aborted Microspores (SlAMS) protein. **a** Amino acid composition. **b** Predicted subcellular location. **c** Secondary structure. **d** Tertiary structure

The predicted tomato SlAMS protein contained 602 amino acid residues: serine (S) was the most abundant residue (9.3%), followed by leucine (E; 7.6%) and arginine (R; 5.5%; Fig. 3a). The protein included 89 negative charges (Asp + Glu) and 64 positive charges (Arg + Lys). Subcellular localization analysis predicted that the tomato SlAMS protein would be confined to the nucleus (Fig. 3b). The putative secondary structure of the protein was comprised of 16.64% α-helixes, 70.04% random coils, and 10.32% extended chains (Fig. 3c and Additional file 2). Consistent with the secondary structure prediction, the predicted tertiary structure of the tomato SlAMS protein was generally ellipsoidal, and primarily composed of random coils (Fig. 3d).

### Pollen viability and morphology of the *SlAMS*-silenced plants

**Fig. 4:**
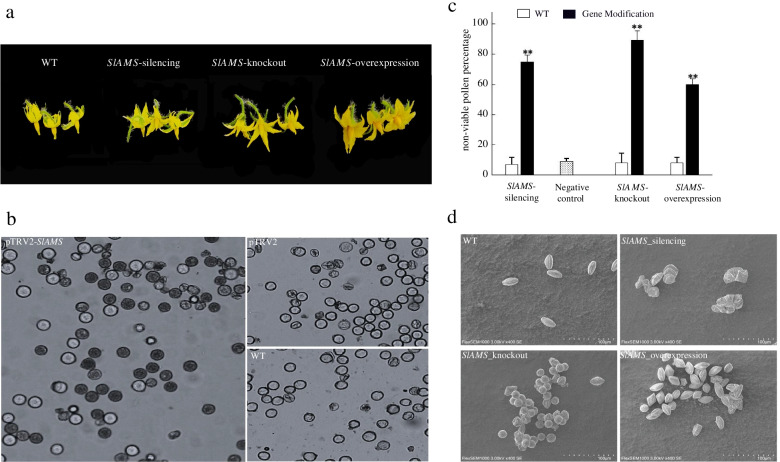
Pollen viability and morphology of the *SlAMS*-silenced tomato plants. **a**. The performance of flowers of *SlAMS*-silenced, −knockouted and -overexpressed plants. **b**. Pollen grain staining after VIGS-mediated gene silencing of *SlAMS*. pTRV2-*SlAMS* transformed; pTRV2 basic vector transformed (negative); Untransformed (WT). Blue-stained pollen grains are non-viable; colorless pollen grains are viable. **c**. The non-viable pollen grains percentage of *SlAMS*-silenced, −knockouted and -overexpressed plants and their corresponding wild type plants. **represents significance difference at 0.01 level (*P* < 0.01). Data are shown as means±SD (*n* = 3). **d**. Scanning electron microscope (SEM) images of pollen grains of *SlAMS*-silenced, −knockouted and overexpressed plants. Untransformed wild type (WT, oval). *SlAMS* silencing (showing tetragonal and sunken shapes). *SlAMS* knockout (showing round or shriveled shapes, from CR-T0–5). *SlAMS* overexpression (showing rhombic and other deformities, from OV-T0–3)

*SlAMS* clones were identified using colony PCR (Additional file 3) and sequenced. The group of albefaction tomato plants exhibited the characteristic white leaf phenotype at 2 or 3 weeks after pTRV2-*PDS* infection (Additional file 4 Fig. S4b), indicating that the VIGS was successful implemented. The states of blooming in silencing plants were generally normal (Fig. 4a; Additional file 5). In the *SlAMS*-silenced plants (infected with pTRV2-*SlAMS* (Additional file 4 Fig. S4a), pollen grains were 75% blue-stained and poorly developed (Fig. 4b, pTRV2-*SlAMS*), suggesting that only 25% pollen grains were viable. In contrast, pollen grains from the wild type (uninfected; Fig. 4b, WT) and negative control plants infected by pTRV1 and pTRV2 (Additional file 4 Fig. S4d) were mostly colorless and thus likely viable, whose non-viable pollen grains proportions were rarely 7 and 9% (Fig. 4b, pTRV2 and WT). The ratio of non-viable pollen grains of *SlAMS*-silenced plants (75%) were extremely significantly higher than that of the wild type and negative control plants (*P* < 0.01; Fig. 4c), Pollen grains staining results also showed that the pTRV2 virus carrier had little effect on pollen activity. After *SlAMS* gene silencing, the activity of the tomato pollen decreased significantly. Under the SEM, the pollen grains from the *SlAMS-*silencing plants appeared irregular shape (tetragonal, sunken and shriveled), while the wild type plants performed oval and plump (Fig. 4d, WT).

### Pollen viability and morphology of *SlAMS*-knockouted plants

**Fig. 5:**
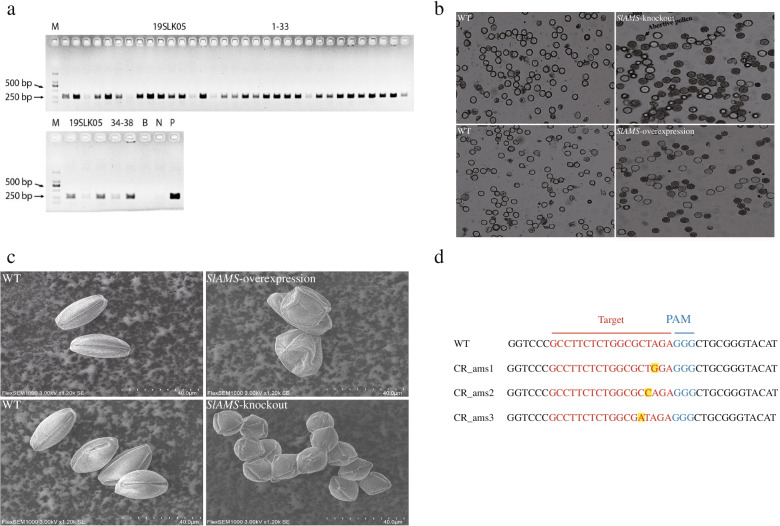
Pollen viability and morphology of tomato plants in different *SlAMS* modification modes. **a**. PCR Identification of positive plants transformed with the pCRISPR/Cas9-*SlAMS* vector. Lanes 1–38: PCR products of *Npt* II; Lane B: no target DNA; Lane N: nontransgenic plant; Lane P: pCRISPR/Cas9-*SlAMS* plasmid. **b**. Pollen viability test after *SlAMS* knockout (CR-T0–2) and overexpression (OV-T0–10). Blue-stained pollen grains are non-viable; colorless pollen grains are viable. **c**. Scanning electron microscope (SEM) images of tomato pollen grains. Wild type (WT, oval shape); pCRISPR/Cas9-*SlAMS* (showing shrinkage and diamond-like shapes, from CR-T0–2). pCAMBIA2301-*SlAMS* (showing shriveled and atrophic shapes, from OV-T0–10); **d**. Target site mutation examination in CRISPR/Cas9-mediated *SlAMS* plant. Target shows the editing site sequence. PAM indicates the adjacent motif of the protospacer sequence. CR-ams1 indicated the first type of mutation; CR-ams2 indicated the second type; CR-ams3 indicated the third type

After the successful construction of the knockout vector CRISPR/Cas9-*SlAMS* confirmed by sequencing, tomato genetic transformation was performed using *Agrobacterium*-mediated methods. The transformation procedure using cotyledon as explants included pre-culture, *Agrobacterium* infection, co-culture, selection culture, regenerating and rooting culture. In total, 38 regenerated plants were obtained using selection medium supplemented with kanamycin. Among them, 33 plants were identified as positively transformed via the PCR identification of the kana-resistance gene *Npt* II (Fig. 5a). To identify mutations of *SlAMS* gene in these positively transformed plants, we conducted sequencing of amplification products of the *SlAMS* fragments, the results indicated that 21 mutant out of 33 *Npt* II positive plants were identified as mutants, thus the frequency of mutations was about 63.6%. The types of *SlAMS* editing in mutant plant were almost single base mutation, including the 16th base A of target sequence mutated to G, the 15th base T to C, and 14th base C to A (Fig. 5d). Previously study reported that the frequency deletion correlated with the target site in tomato via CRISPR/Cas9 system, the ratio of 1 to 10 bp deletion was about 67%, but only 3% for 10 bp or higher deletion [28]. It was obvious that shorter deletions such as 1 bp appeared at high frequency in our system.

Despite the *SlAMS*-knockout plants blossomed normally in anthesis (Fig. 4a; Additional file 5 Fig. S5c), the pollen grains were poorly developed, and about 89% pollen grains were stained blue (Fig. 5b, *SlAMS*-knockout). This indicated that only 11% of the grains were viable. In the untransformed wild type plants, pollen viability proportion was 92% (Fig. 5b, WT). The percentage of non-viable pollen grains of *SlAMS* knockout plants (89%) were extremely significant higher than that of the wild type plants (*P* < 0.01; Fig. 4c). Under the SEM, the pollen grains from the *SlAMS-*knockout plants appeared round, diamond-like or shrinkage (Fig. 4d; Fig. 5c, *SlAMS*-knockout). In contrast, pollen grains from the wild type plants appeared oval (Fig. 4d; Fig. 5c). Thus, both pollen staining and SEM observation indicated that *SlAMS* knockout remarkably reduced pollen viability as compared to the wild type plants.

### Pollen viability and morphology of *SlAMS-*overexpressed plants

PCR verification of the positive colonies indicated that the target amplicon was about 1800 bp in length (Additional file 6). After implementing *Agrobacterium-*mediated transformation (Additional file 7), PCR detection of the *Npt* II gene showed that 17 plants had been successfully transformed with pCAMBIA 2301-*SlAMS* (Additional file 8).

The characters at florescence stage in the *SlAMS-*overexpressed plants were shown in Fig. 4a and Additional file 5 Fig. S5d. In viability test, pollen grains were poorly developed, and about 60% were stained blue. This indicated that merely 40% of the pollen grains were viable, while the proportion in wild type plants were 91% (Fig. 5b). The ratio of non-viable pollen grains of *SlAMS*-overexpressed plants (60%) were notably higher than that of the wild type plants (*P* < 0.01; Fig. 4c). Under the SEM, the pollen grains from the *SlAMS*-overexpressed plants were rhombic, atrophic, shriveled, or otherwise abnormally shaped (Fig. 4d; Fig. 5c). Thus, both pollen staining and SEM observation indicated that *SlAMS* overexpression considerably reduced pollen viability as compared to the wild type plants. See Additional file 16 for all unedited gel pictures in the paper.

### qRT-PCR analyses of *SlAMS*

**Fig. 6:**
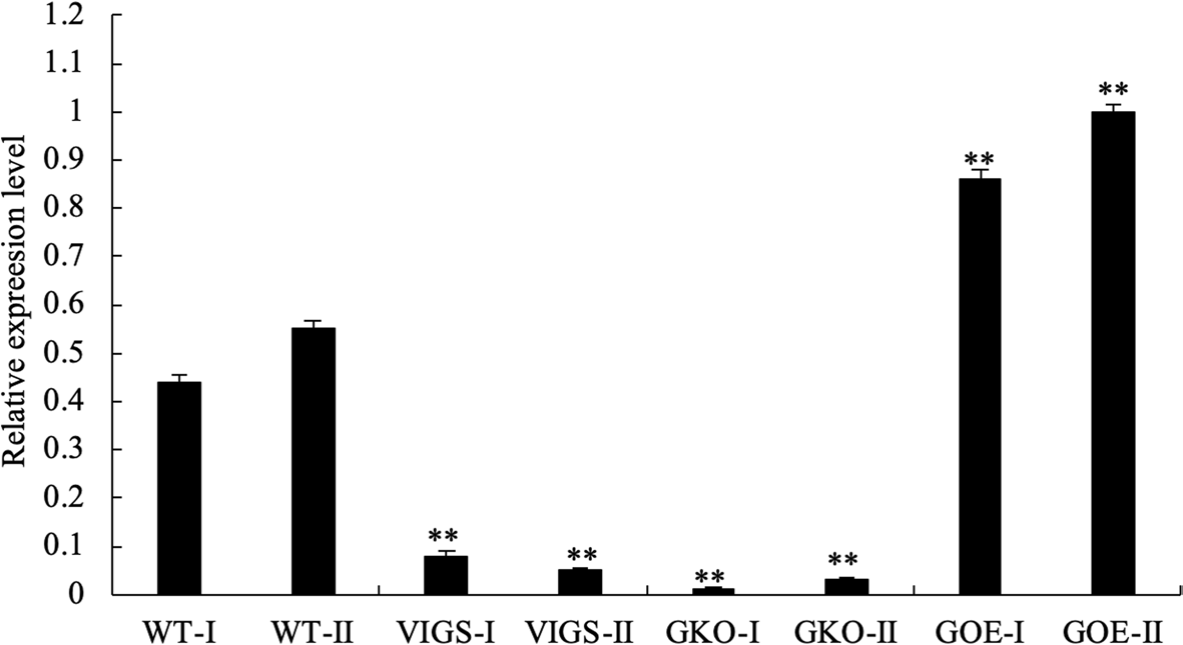
Relative expression analysis of *SlAMS-*silenced, −knockouted and -overexpressed tomato plant by qRT-PCR at tetrad and maturity stage. WT represents wild type anther. VIGS represents *SlAMS* silencing anther, GKO represents *SlAMS* knockouting anther, GOE represents *SlAMS* overexpression anther. Suffix ‘I’ and ‘II’ indicate tetrad stage and maturity stage respectively. Data are presented as means±SD (*n* = 3), **indicates significant differences at *P* < 0.01

We performed qRT-PCR using RNA from *SlAMS*-silenced, −knockouted, −overexpressed anthers to verify whether those biotechnological system would be effective, and further if the down or upregulation of *SlAMS* affect pollen development in tomato. The qRT-PCR analysis showed that, both at tetrad and maturity stage, the expression levels of *SlAMS* in silencing and knockout anthers reduced significantly (*P* < 0.01) when compared with that of wild type anthers (Fig. 6), thus demonstrated that the *SlAMS* expressions were downregulated after being silenced and knockouted (*SlAMS*-knockout plants conferred the lower expression level), and as a result, the abnormal pollen development occurred and abortive pollen produced in those plants. But noticeably, the qRT-PCR results appeared *SlAMS* had higher expression in overexpressed anthers contrasted to in that of wild type anthers (*P* < 0.01), the upregulation of *SlAMS* also affected the pollen formation and led to the decreasing of viability. Combined the results of qRT-PCR, pollen viability and morphology assessments, the compound results suggested that *SlAMS* might be essential for regulating pollen development in tomato.

## Discussion

Although pollen development is an extraordinarily complicated process, several transcription factors that regulate tapetum development have been cloned and their function identified, including *DYT1*, *TDF1*, *AMS* and *MS188* [18, 20, 22, 29]. Here, we cloned a candidate tomato gene, *SlAMS* (1806 bp), which may be essential for tapetum and pollen development and result in male sterility in tomato. Our phylogenetic tree indicated a close evolutionary relationship among the SlAMS proteins and other plants, suggesting that SlAMS may be generally conserved within families, especially conserved across this group of the Solanaceae (e.g., *S. pennellii,* potato*,* and pepper). The SlAMS protein was also located in the nucleus and was primarily composed of irregular curls, implying that SlAMS acts as a transcription factor, regulating the expression of nuclear genes. The cloned *CaAMS* from *Capsicum annuum* also encodes a bHLH transcription factor and is localized to the nucleus, it seems to play an crucial role in pollen and tapetum development in pepper by regulating a complex network [26].

Due to the emasculation procedure of manual removing anthers, the costs of F1 seed production will be raised in major horticultural crops such as tomato and pepper, however, the utilization of male sterility is a very effective approach to solve the hybrid seed costs problem and ensure the high purity. Tomato male sterility has been an interest for current researchers. To date, the application of male sterility in cross breeding of tomato has not been successfully carried out for the mutants are nearly spontaneous, which requires a long period to eliminate linkage drags in backcrossing process. Besides the reported male-sterile mutants of *MS32* and *MS10*^*35*^ [30, 31], recent researchers have demonstrated that male sterility in tomato plant was acquired after modifying of the *SlMS10* gene via CRISPR/Cas9-mediated system, which could be introduced into the elite lines [28]. It was considered that the *SlMS10* and *MS32* were the upstream genes of *AMS-like* genes involved in pollen and tapetum development, the loss function of these genes resulted in downregulation of the other transcription factors gene such as *AtTDF1-like*, *AtAMS-like*, *AtMYB-like* and *AtMS1-like* in tomato [28, 31]. Similarly, the downregulation of the *CaAMS* gene by VIGS system also led to male sterility in pepper and melon [26]. In this study, we demonstrated that alternative approaches to generating male sterility in tomato crop by the gene silencing, −knockout and -overexpression of the *SlAMS* gene, of which CRISPR/Cas9-mediated genome editing of *SlAMS* conducted the best performance with the lowest viable pollen ratio (11%). Recently Jung et al. reported that the T1 and T2 generations of elite male-sterile lines, exhibiting abnormal tapetum and producing no pollen, were obtained by knockout of *SlMS10* in tomato [28]. In comparison with the previously work, although we have obtained the pollen abortion-type male-sterile material in tomato, more research work must be conducted in the future. Firstly, as we used variety ‘Ailsa Craig’ in which was successfully established the highly effective genetic transformed system, it is necessary but time-consuming to eliminate linkage drags by executing backcross breeding process to transfer the male sterility trait to the comprehensive parents, moreover further transformation investigation would be done in the elite inbred lines so as to performing CRISPR/Cas9-mediated gene knockout. Secondly, We expected that the *SlAMS*-knochouted plants would have the most valuable application prospects for the highest proportion of non-viable pollen. Nevertheless, pollens of the knockout plants were not entirely abortive, 11% pollens still fertile. This may lead to pseudohybrids if utilized in hybrid seed production, hence the CRISPR/Cas9-mediated system should be optimized such as editing site selection, and the hybrid process should be done to assess the practical application value and prospects in hybrid breeding in tomato.

It was clearly reported that two conserved pathway, namely, the *DYT1-TDF1-AMS-bHLH10/89/91-MYB80* transcription cascade in *Arabidopsis* and the *UDT1-TDR1-TIP2-EAT1* in rice [31, 32]. With regard to the *AMS* gene, it has been cloned in *Arabidopsis* and other model plants, and the mechanisms by which *AMS* controls anther development in *Arabidopsis* have been well studied [16]. Indeed, previous studies have demonstrated that *AMS* plays a central role in the coordination of sporopollenin biosynthesis and the secretion of materials for pollen wall patterning [33], and the *AMS-like* gene is requried for anther and microspore development in pepper [26]. However, the role of the *SlAMS* gene in the regulatory network controlling pollen and tapetum development in tomato has not yet been investigated so far. Ferguson et al. reported that, in *Arabidopsis*, the biphasis expression was essential for tapetum and pollen formation, along with an initial peak around pollen meiosis and then later during pollen wall development, and AMS protein may competitively form a protein complex with other transcription factors, causing the repression of upstream regulators and promotion protein degradation, the researchers also indicated that additional factors are associated with the *AMS* regulation [24]. Noticeably, in this paper, the over-expression of *SlAMS* gave rise to the upregulation of *SlAMS* both at tetrad and maturity stages, and thus affected pollen formation and led to the decreasing of pollen activity in tomato plants. We deduced that the *SlAMS* upregulation might competitively repress the expression of upstream transcription factors such as *MS*, *DYT1* or *TDF1*. It was indicated in our study that the gene silencing, knockout and over-expression of the tomato *SlAMS* gene strongly affected the its expression levels, and subsequently resulted in the morphological deformation of the pollen grains and pollen viability reduction, it is likely that *AMS* plays a crucial role in tapetal cell development and the post-meiotic transcriptional regulation of microspore development within the developing anther [16]. However, the expressions of other involved genes should be examined by qRT-PCR or RNA-seq in *SlAMS-*upregulated and downregulated plants to confirm the underlying pathway regulating the pollen and tapetum development.

In summary, in this paper, we only cloned the tomato *SlAMS* gene and explored the possibility of creation the novel male-sterile materials in tomato by *SlAMS* gene silencing, knockout and overexpression technology. Our results have laid a fundamental foundation for follow-up studies investigating the *SlAMS*-associated molecular mechanism regulating anther formation in tomatoes, as well as the creation of male-sterile tomato lines. Up to now, we have being carried out the further research work and made some progress: the pollen viability is measured in T1 progeny transgenic plants, having used the anther and pollen sample in different developmental stage to conduct transcriptome studies in *SlAMS*-knochouted and -overexpressed plants, we expect that the *SlAMS-*associated regulatory networks and pathways regulating pollen development and pollen fertility would be clarified in further studies.

## Conclusion

To reduce the cost of hybrid seed production and ensure high purity, it is important to utilize the male sterility in crossbreeding of tomato. In this study, the *SlAMS* gene from tomato pollen cDNA was isolated and characterized. The sequencing results indicated that the tomato *SlAMS* gene had the length of 1806 bp encoding 602 amino acid residues. In addition, bioinformatics analysis showed that *SlAMS* gene shared the higher sequence similarity (85% ~ 98%) and exhibited a close evolutionary relationship with other known *AMS* in Solanaceae speices. Subsequently, by the use of VIGS, CRISPR/Cas9 and overexpression biotechnology, the proportion of non-viable pollen grains in *SlAMS*-silenced (75%), −knockouted (89%), and -overexpressed (60%) tomato plants were extremely significant higher than that of the untransformed plants (*P* < 0.01). The morphology performances in three cases of non-viable pollen grains comprised tetragonal, circular, atrophic, shriveled, or otherwise abnormally shaped, while pollen grains of the wild type plants appeared oval and plump. The qRT-PCR results indicated that the expression levels in anthers of *SlAMS*-silenced and -knockout plants were extremely lower than that of wild type plants, yet *SlAMS* gene had higher expression level in anthers of of *SlAMS-*overexpressed plants.

Thus, we demonstrated that the gene-silencing, −knockout and -overexpression of the *SlAMS* gene in tomatoes induced the *SlAMS* downregulation or upregulation, and this may lead to abnormal pollen development, which in turn decrease pollen viability, and subsequently generate male-sterile lines. Our research suggested alternative approaches to generating male sterility in tomato crop, among them, CRISPR/Cas9-mediated genome editing of *SlAMS* implied the best performance.

## Materials and methods

### Plant materials

Tomato (*Solanum lycopersicum* L.) plants of cv. ‘Ailsa Craig’ were grown in a greenhouse at Yunnan Agricultural University (Kunming, China). Greenhouse temperature was maintained at 26 °C during the day (16 h) and at 18 °C during the night (8 h). Tomato flower buds were removed from the plants at flowering period and placed in an ice box. Anthers were collected under low temperatures, quickly treated with liquid nitrogen and stored at − 80 °C.

### Cloning and characterization of the tomato *SlAMS* gene

Total RNA was extracted from tomato anthers using Trizl Kits (TransGen Biotech. Co., Ltd., Beijing, China). The cDNA of the tomato was synthesized using High Fidelity Prime Script RT-PCR Kits (Takara Biomedical Technology Co., Ltd. Beijing, China), following the manufacturer’s instructions, and amplified using PCR with the primers *AMS*ad1 and *AMS*ad2 (Additional file 13). Primers were designed based on the previously-published tomato *AMS* gene sequence (GenBank ID. LOC101253608) using Primer 5.0. The PCR volume contained 2 μL cDNA, 1 μL each primer (10 μmol/μL), 12.5 μL of 2 × EasyTaq PCR SuperMix, and ddH_2_O to make 25 μL. The PCR cycling conditions were as follows: pre-denaturation at 95 °C for 3 min; 35 cycles of denaturation at 94 °C for 50 s, annealing at 50 °C for 1 min, and extension at 72 °C for 3 min; a final extension at 72 °C for 10 min; and an indefinite hold at 4 °C. The target DNA fragment was extracted from the agarose gel using by gel filtration, and the purified using a TIANgen Midi Purification Kit (TIANGEN Biotech. Co., Ltd., Beijing, China) following the manufacturer’s instructions. The target fragment was ligated to the pMDT-18 cloning vector (0.5 μL of vector, 4 μL of PCR product, 5 μL of Solution I, and 0.5 μL of T4 DNA ligase) to form pMDT-18-SlAMS, incubated at 16 °C for 3 h, and then transformed into *Escherichia coli* DH_5α_ competent cells. The transformed cells were cultured overnight at 37 °C. Six single white colonies were selected, and the length of the inserted fragment was confirmed using PCR. Recombinant plasmid DNA was isolated using TIANprep Mini Plasmid Kits (Yunnan Morning Green Biotechnology Co., Ltd. Yunnan, China) and sequenced by the Shuo Qing Biotechnology Co., Ltd. (Kunming, China).

The homology of the cloned *SlAMS* gene sequence was investigated using DNAMAN 6.0 and NCBI BLAST. To compare the predicted tomato SlAMS protein with other AMS homologs, both within the Solanaceae and among other closely-related families (i.e., the Compositae, Cruciferous, Leguminosae, and Cucurbitaceae), we constructed a phylogeny of the AMS proteins from 25 plant species (Additional file 14) using the neighbor-joining method in MEGAX10.1.5. A subcellular localization analysis was performed using PSORT II predict. The primary structure of the encoded SlAMS protein was predicted using PredictProte; the secondary structure was predicted using SOPMA; and the tertiary structure was predicted using SWISS-MODEL. The websites for the online tools are given in Additional file 15.

### Obtaining the *SlAMS-*silenced plants by VIGS and morphological observation of pollen

We designed the primers *AMS*-F and *AMS*-R (Additional file 13) to amplify the *SlAMS*-interference fragment which was 274 bp long (Additional file 1), using the SNG VIGS TOOL (https://vigs.solgenomics.net/) and pTRV2 as a basic VIGS vector (Additional file 9), we amplified the interference fragment from cloning vector pMDT-18-SlAMS as a template. We chose tobacco brittle virus (TRV) as gene silencing vector [34, 35]. The TRV genome contains two RNA chains, RNA1 and RNA2, which form binary vectors. TRV vector-mediated gene silencing requires the simultaneous action of the RNA1 and RNA2 chains [36]. The pTRV2 vector was double-digested with *Sac* I and *Bam* HI, and then ligated to the *SlAMS-*interference fragment to form the pTRV2-*SlAMS* carrier. The recombinant *SlAMS* products were then transformed into *E. coli* DH_5α_ and *Agrobacterium tumefaciens* GV310. *SlAMS* clones were verified using colony PCR with the primers *AMS*-R and pTRV2-seqE (Additional file 13) and then sequenced. The positive clones were fully mixed with 50% glycerin (a glycerin: bacterial culture ratio of 1:1) and stored at − 80 °C.

Tomato plants were randomly selected and allocated among four groups: plants in the silencing group were infected with *A. tumefaciens* carrying pTRV2-*SlAMS* and pTRV1; plants in the albefaction group (mock-infection) were infected with *A. tumefaciens* carrying pTRV2-PDS and pTRV1; plants in the negative control group were infected with *A. tumefaciens* carrying pTRV2 and pTRV1; and plants in the blank control group were uninfected wild type plants. Each group was represented by three replicates, and each replicate was comprised of 10 plants. To activate *Agrobacterium*, 20 μL of glycerol-stored *Agrobacterium* carrying pTRV2-*SlAMS,* pTRV1, pTRV2, or pTRV2-*PDS* were added to 1 ml of Luria-Bertani medium (supplemented with 50 μg/ml kanamycin and 25 μg/ml rifampicin), and incubated overnight at 28 °C with shaking at 200 rpm. After incubation, the bacterial solution was added to 10 ml of LB medium and cultured overnight at 28 °C with shaking at 200 rpm. The bacterial solution was then centrifuged at 800 g for 15 min. Each *A. tumefaciens* pellet was resuspended in sufficient infection solution to make an optical density (OD) at 600 nm of 1.0. Then, each *A. tumefaciens* solution (carrying pTRV2-*SlAMS,* pTRV2, or pTRV2-*PDS*) was separately mixed with pTRV1 at a volume ratio of 1:1. The *A. tumefaciens* mixtures were cultured at 28 °C with shaking at 50 rpm for 3 h. After two euphyllas had completely unfolded, 30 plants per group were injected with 1 ml of the appropriate *A. tumefaciens* solution; the plants in the blank control group were injected with sterile water. After injection, plants were incubated in the dark at 21 °C for 24 h, and then cultivated in the greenhouse, covered with nylon nets to avoid cross-contamination and insect-mediated virus transfer, until flowering.

Pollen was collected from five flowers from each plant in the wild type, negative control, and *SlAMS*-silenced groups. Pollen viability was measured using the blue-ink staining method. In brief, a small amount of pollen was placed on a glass slide, and a drop of distilled water was added. The pollen grains were spread out with tweezers, and then a drop of blue ink solution was added. Viable and non-viable pollen grains were counted in three different fields of view, and the final percent viable was calculated as the average of the three counts; unstained pollen were viable, while blue-stained pollen were abortive. The significance analysis of non-viability pollen percentage was implemented using *t* test method by statistical software SPSS 25.0. The morphological characters of the pollen grains were then observed under a scanning electron microscope (SEM) with an acceleration voltage (EHT) of 3.0 kv and a magnification (MAG) of 1.2 k.

### pCRISPR/Cas 9-mediated *SlAMS* knockout and mutation analysis

A pCRISPR-*SlAMS* vector was constructed using a Cas9/gRNA plasmid construction kit (VK005–08, Beijing Viewsolid Biotech., Co., Ltd., Beijing, China), following the manufacturer’s instructions. In brief, the target gRNA sequence was designed based on the cloned *SlAMS* gene sequence using an online CRISPR design tool (crisPr.mit.edu). We then designed sense and anti-sense primers (pCRISPR-Sens and pCRISPR-Anti, respectively; Additional file 13) based on the target sequence and synthesized the oligomer. The oligomer was inserted into the Cas9/gRNA vector and transformed into *E. coli* DH_5α_ (Additional file 10). Then, colonies were randomly selected and sequenced to identify positive clones using the primers pCRISPR-SeqE (Additional file 13). The identified pCRISPR-*SlAMS* vector was transformed into *A. tumefaciens* GV3101. Then, we used *Agrobacterium*-mediated method to transform the vector into tomato genome [37]. We conducted three batches of *Agrobacterium* infection using cotyledons (i.e 3 replicates), which comprised 80 ~ 100 explants in each batch.

After the co-culture, selection culture of the infected cotyledons, the regenerated plants were obtained. The genomic DNA were extracted from T0 progeny regenerated plants using the CTAB method. Regenerated but untransformed plants were used as controls. We detected transgenic plants via the PCR amplification of the *Npt* II selection gene. The PCR volume included 1 μL DNA; 2 μL 10 × PCR buffer, 0.4 μL dNTP mixture (2 mmol/L each), 0.2 μL forward and reverse primers (10 μmol/L), 0.2 μL Taq DNA polymerase (1 U/μL); and ddH_2_O to make 20 μL. The thermal cycling procedure was as follows: 94 °C for 3 min; 30 cycles of denaturation at 94 °C for 30 s, annealing at 58 °C for 30 s, and extension at 72 °C for 30 s; and a final extension at 72 °C for 10 min. In the CRISPR/Cas9 system, the Cas cleavage site occurs mostly 3 bp upstream of the protospacer, thus the insertion and deletion mutations around 3 bp upstream of the protospacer were considered to be mutations induced by Cas9. To identify the mutations, we designed the primers (AMS-F2/AMS-R2, Additional file 13) based on the gene editing site of *SlAMS*, and extracted the flower DNA of 33 individual *Npt* II positive plants. DNA extraction and PCR cycling conditions were the same as the *Npt* II ampification. The 267 PCR products were sequenced and confirmed for mutation (ShuoQing Biotech., Co., Ltd., Kunming, China).

The positive transgenic plants and the untransformed wild type plants were then cultivated in the greenhouse until flowering (covered in a nylon net to avoid cross-contamination as above). Pollen viability ratios in the control and transgenic plants were assessed as described above. The methods of statistic analysis and morphological observation of pollen grains were conducted as described above.

### *Agrobacterium*-mediated *SlAMS* overexpression

Primers 2301-AMSF and 2301-AMSR (Additional file 13) were designed to amplify the *SlAMS* gene, using clone vector pMDT-18-*SlAMS* as a template. The *SlAMS* target gene fragment was ligated to the pCAMBIA2301 vector after digestion with *Sac* I and *Bam* HI to construct the overexpression vector pCAMBIA2301-*SlAMS* (Additional file 11). The recombinant product was transformed into *E. coli* DH_5α_ and *A. tumefaciens* GV3101. After verification of a single colony using PCR and sequencing, the positive clones were fully mixed with 50% glycerin (a glycerin: bacterial culture ratio of 1:1) and stored at − 80 °C.

*Agrobacterium*-mediated transformation was performed using cotyledons as target infection explants as described above. Three batches of *Agrobacterium* infection using cotyledons were conducted. The transgenic plants were then cultivated until flowering as described above. The genomic DNA of T0 progeny transformed plants were extracted by CTAB method and then detected transgenic plants via the PCR amplification of the *Npt* II gene contained in the overexpression vector pCAMBIA2301. Pollen viability and morphology were compared among the positive transgenic plants and the control plants as described above. The methods of statistic analysis and morphological observation of pollen grains were the same as above.

### Anther RNA extraction and qRT-PCR analyses of *SlAMS*

We collected the anthers of *SlAMS-*silenced, −knockouted, −overexpressed plants and wild type plants during the flowering period at two pollen formation stages (tetrad and maturity). Total RNA was isolated from anthers of two flower buds using Trizol Kits (TransGen Biotech. Co., Ltd., Beijing, China) following the manufacture’s instructions, and the cDNA was synthesized via performing the reverse transcription as described in the cloning of *SlAMS* gene. For *SlAMS* gene expression level detection, the specific primers (qAMS-F/qAMS-R) for qRT-PCR were designed based on the cloned *SlAMS*. We used *Actin* (GQ337966.1) as the internal control gene for its stable expression level in different plant tissues (Actin-F/Actin-R). The primers used were listed in Additional file 13.

The qRT-PCR was conducted using TB Green® PrimeScript™ RT-PCR Kit (TaKaRa Bio. Co., Beijing, China) following the manufacture protocol on ABI 7500 real-time PCR system (Applied Biosystems, USA). The 10 μL qRT-PCR volume contained 0.2 μL each primer (10 μL mol/μL), 5 μL of 2XTB Green RT-PCR Buffer, 1 μL cDNA (200 ng), 3.6 μL ddH_2_O. Cycling reaction conditions were 95 °C for 3 min, followed by 40 cycles of 10 s at 95 °C, 15 s at 60 °C, and 20 s at 72 °C. Three biological replicates (at least three plants for each replicate) were performed in qRT-PCR. The relative expression levels of *SlAMS* were calculated by the 2^−△△Ct^ method. The difference significant analysis was conducted via *t* test using statistic software SPSS 25.0.

## Supplementary Information

**Additional file 1: Fig. S1.** Sequence of the tomato *SlAMS* gene and the deduced protein sequence. The region highlighted in yellow was used for the VIGS experiment.

**Additional file 2: Fig. S2.** The predicted secondary structure of the tomato SlAMS protein.

**Additional file 3: Fig. S3.** PCR detection of colonies carrying pTRV2-*SlAMS* and positively transformed tomato plants.

**Additional file 4: Fig. S4.** VIGS-mediated silencing of *SlAMS* in tomato plants. **a** Infected with *A. tumefaciens* carrying pTRV2-*SlAMS* + pTRV1. **b** Infected with *A. tumefaciens* carrying pTRV2-*PDS* + pTRV1. **c** Negatively infected with *A. tumefaciens* carrying the basic vector pTRV2 + pTRV1. **d** Uninfected wild type.

**Additional file 5: Fig. S5.** The performance of inflorescence of *SlAMS*-silenced, −knockouted and -overexpressed tomato plants. **a** Untransformed wild type. **b** *SlAMS*-silenced plant. **c** *SlAMS*-knockouted plant. **d** *SlAMS*-overexpressed plant.

**Additional file 6: Fig. S6.** PCR identification of the overexpression vector pCAMBIA2301-*SlAMS*. Lane A: 5 k Marker; Lane 1: -CK; Line2: *SlAMS* gene amplification products.

**Additional file 7: Fig. S7.** The *Agrobacterium-mediated* transformation of pCAMBIA230-*SlAMS* vector using tomato cotyledons. **a** pre-culture of cotyledons; (**b**) selection and regenerated culture; (**c**) seedling culture producing cotyledons; (**d**) transgenic plants transplanting after rooting culture.

**Additional file 8: Fig. S8.** PCR identification of tomato plants positively transformed with the pCAMBIA2301-*SlAMS* vector. Lanes 1–31: PCR products of *Npt* II; B: no target DNA; Lane N: nontransgenic plant; Lane P: pCAMBIA2301-*SlAMS* plasmid.

**Additional file 9: Fig. S9.** The map of pTRV2 vector used for the virus induced gene silencing (VIGS) of the tomato *SlAMS* gene.

**Additional file 10: Fig. S10.** The map of pCRISPR/Cas9- *SlAMS* vector used for the tomato *SlAMS* knockout.

**Additional file 11: Fig. S11.** The map of pCAMBIA2301-*SlAMS* vector used for the tomato *SlAMS* overexpression.

**Additional file 12: Table S1.** The *AMS* gene sequences in Genebank used for nucleotide similarity analysis compared with the cloned *SlAMS* gene from tomato.

**Additional file 13: Table S2.** Primer sequences used in this paper (designed using Primer 5.0).

**Additional file 14: Table S3.** Accession numbers of the AMS proteins used in the phylogenetic tree.

**Additional file 15: Table S4.** The web sites used for bioinformatics analyses.

**Additional file 16.**

## Data Availability

The data underlying this study are available on NCBI GenBank (Accession No. MK591950.1 and No. QDO73362.1).

## Abbreviations

*AMS*: Aborted Microspores
*MS*: Male Sterility
*VIGS*: Virus Induced Gene Silencing
*PDS*: Phytoene Desaturase
SEM: Scan Electron Microscope
CRISPR: Clustered regularly interspaced short palindromic repeat
