# Supplementary material for: Gene silencing, knockout and over-expression of a transcription factor ABORTED MICROSPORES (SlAMS) strongly affects pollen viability in tomato (Solanum lycopersicum)
Source: BMC Genomics. 2022 May 5;23(Suppl 1):346. doi: 10.1186/s12864-022-08549-x (PMC9069838; doi:10.1186/s12864-022-08549-x)
Supplement: Supplementary file 1 — Additional file 1: Fig. S1. Sequence of the tomato SlAMS gene and the deduced protein sequence. The region highlighted in yellow was used for the VIGS experiment. [file 12864_2022_8549_MOESM1_ESM.docx]

**
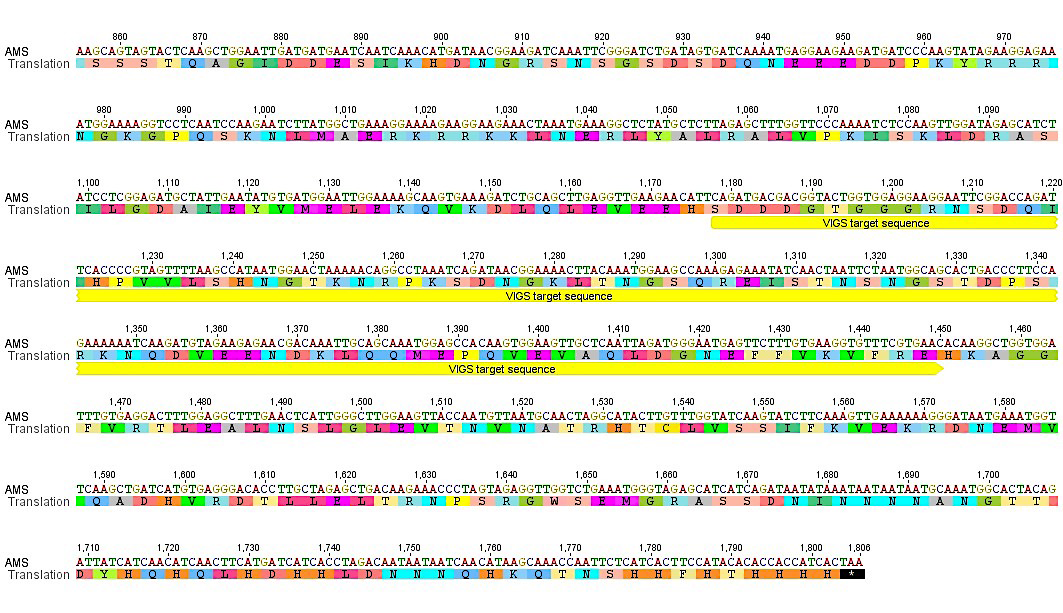
**

**Fig. S1** Sequence of the tomato SlAMS gene and the deduced protein sequence. The region highlighted in yellow was used for the VIGS experiment.
