# Supplementary figures and images for: Gene silencing, knockout and over-expression of a transcription factor ABORTED MICROSPORES (SlAMS) strongly affects pollen viability in tomato (Solanum lycopersicum)
Source: BMC Genomics. 2022 May 5;23(Suppl 1):346. doi: 10.1186/s12864-022-08549-x (PMC9069838; doi:10.1186/s12864-022-08549-x)

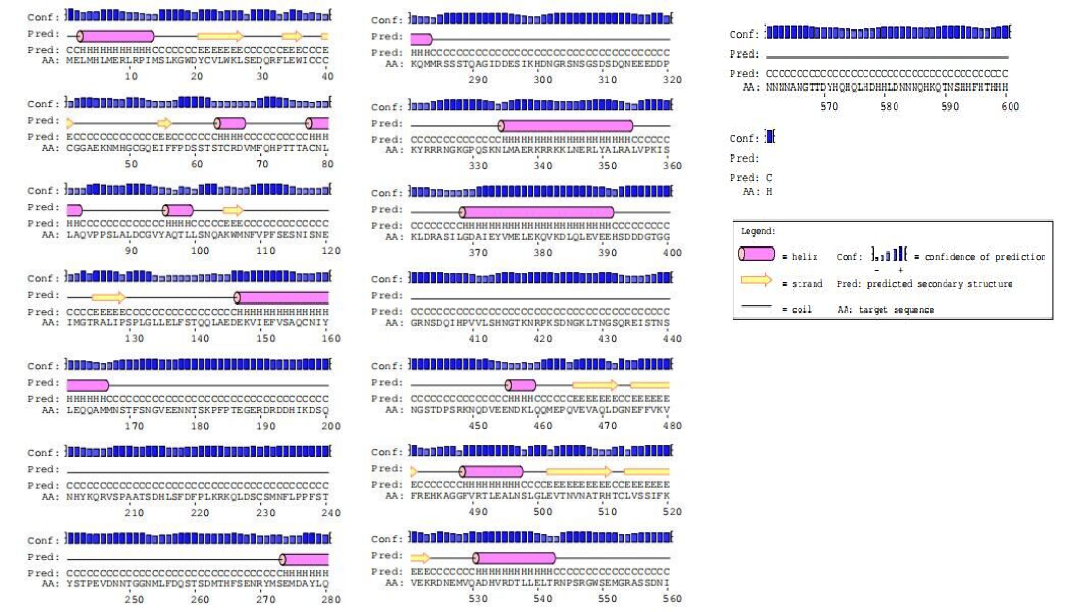


**Fig. S2** The predicted secondary structure of the tomato SlAMS protein.

Supplement: Supplementary file 2 — Additional file 2: Fig. S2. The predicted secondary structure of the tomato SlAMS protein. [file 12864_2022_8549_MOESM2_ESM.docx]

**
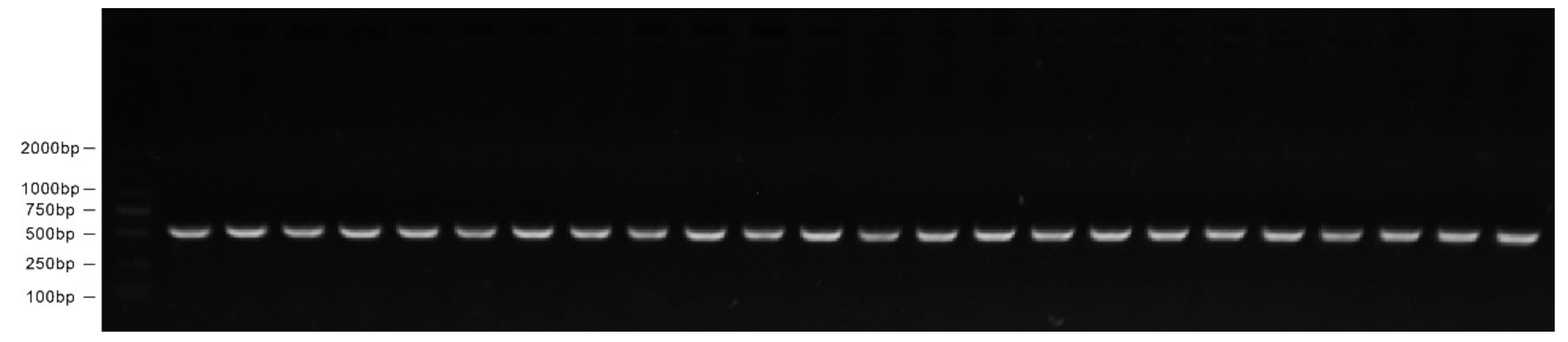
**

**Fig. S3** PCR detection of colonies carrying pTRV2-SlAMS and positively transformed tomato plants.

Supplement: Supplementary file 3 — Additional file 3: Fig. S3. PCR detection of colonies carrying pTRV2-SlAMS and positively transformed tomato plants. [file 12864_2022_8549_MOESM3_ESM.docx]

**
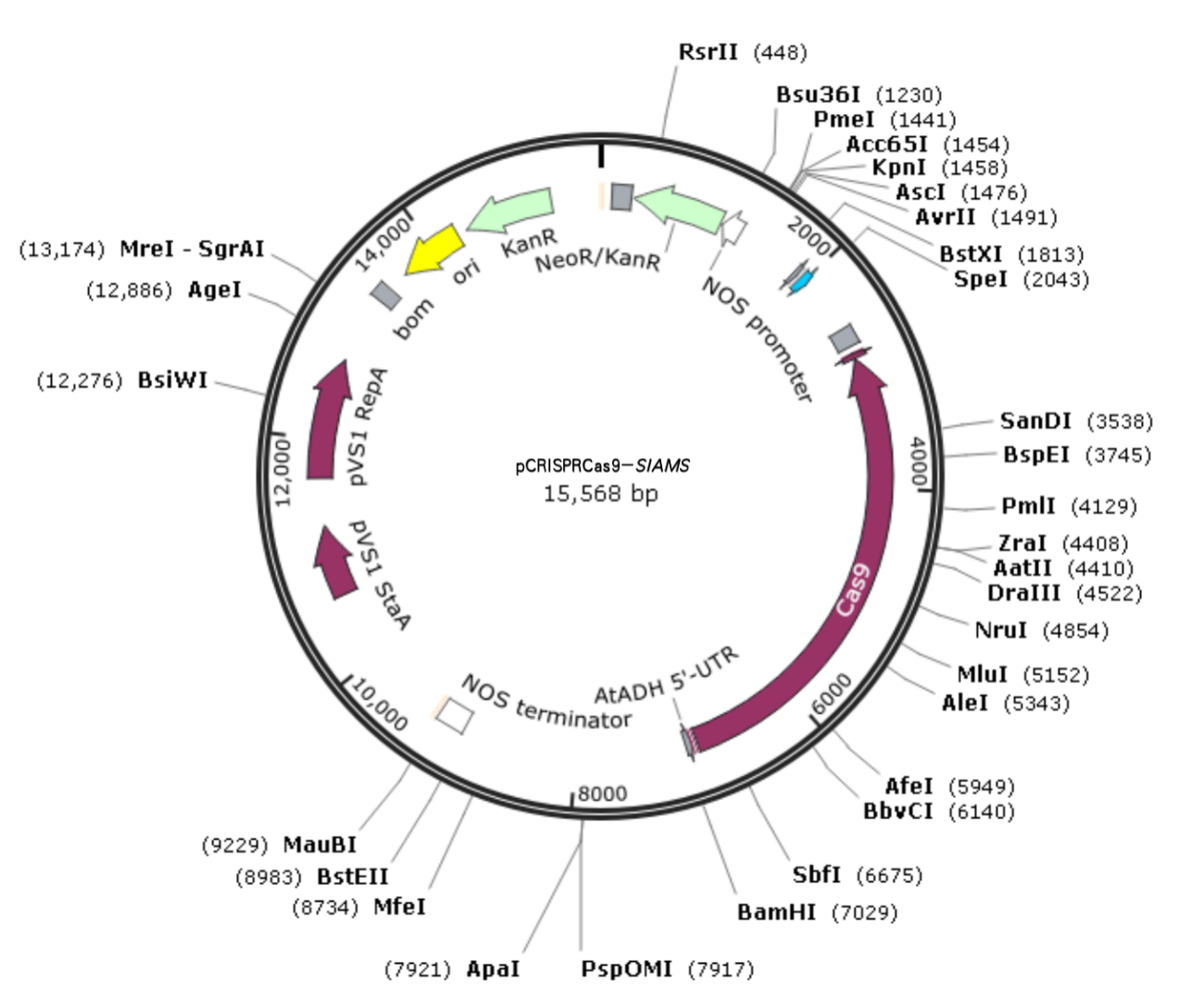
**

Fig.S10 The map of pCRISPR/Cas9- SlAMS vector used for the tomato SlAMS knockout.

Supplement: Supplementary file 10 — Additional file 10: Fig. S10. The map of pCRISPR/Cas9- SlAMS vector used for the tomato SlAMS knockout. [file 12864_2022_8549_MOESM10_ESM.docx]

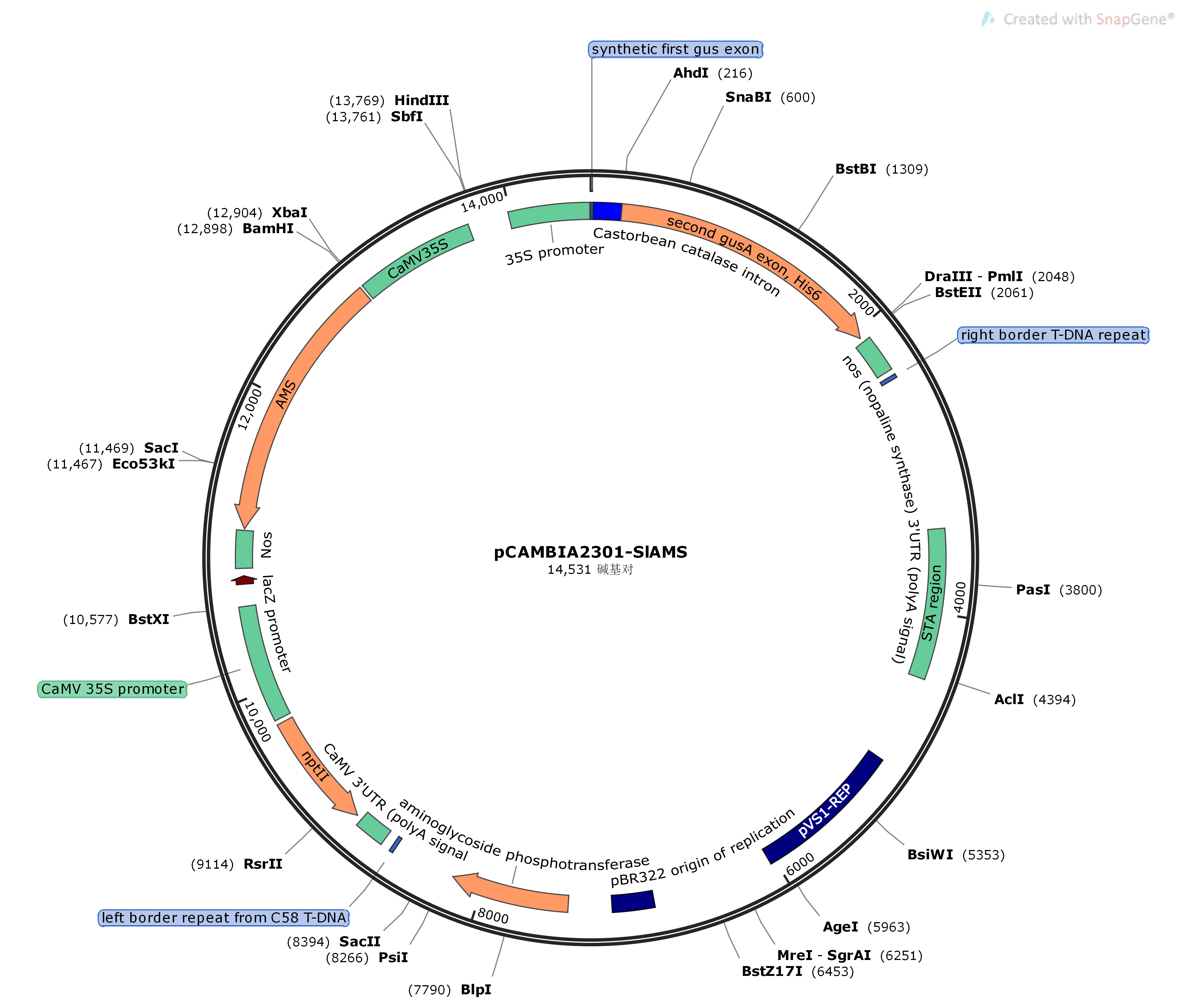


**Fig.S11** The map of pCAMBIA2301-*SlAMS* vector used for the tomato *SlAMS* overexpression.

Supplement: Supplementary file 11 — Additional file 11: Fig. S11. The map of pCAMBIA2301-SlAMS vector used for the tomato SlAMS overexpression. [file 12864_2022_8549_MOESM11_ESM.docx]

**Unedited images of the whole gel blots**


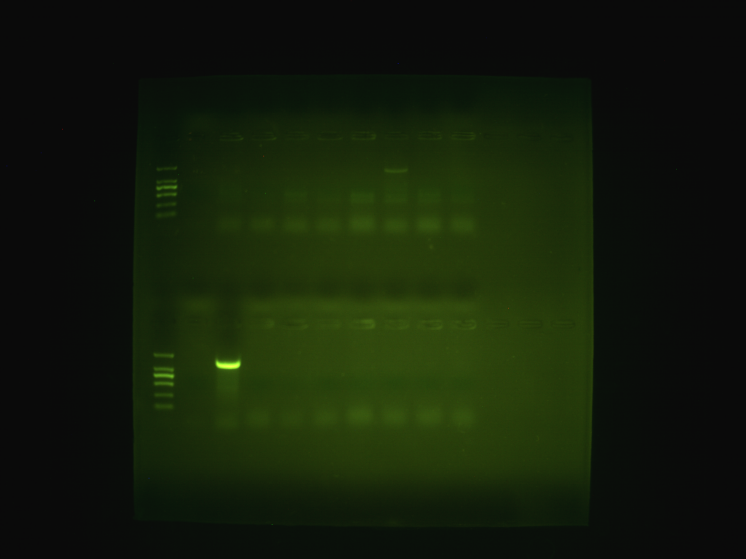


**Fig1a**


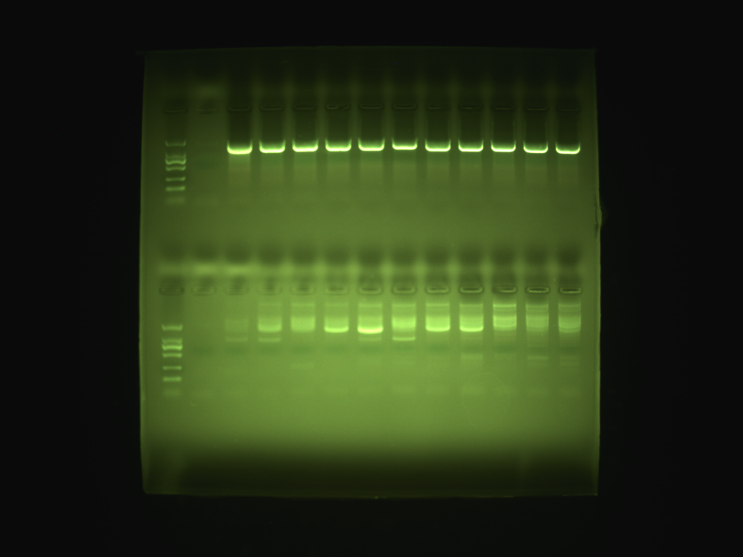


**Fig1b**

**
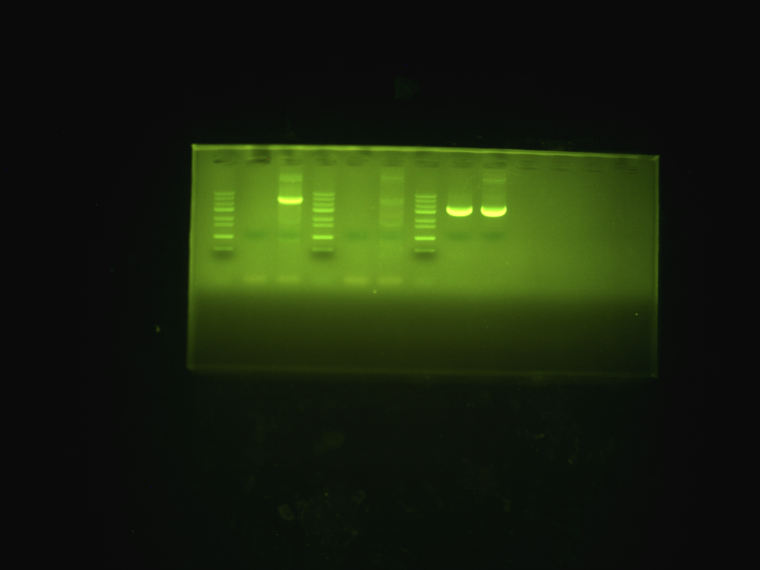
**

**Fig. S6**

**
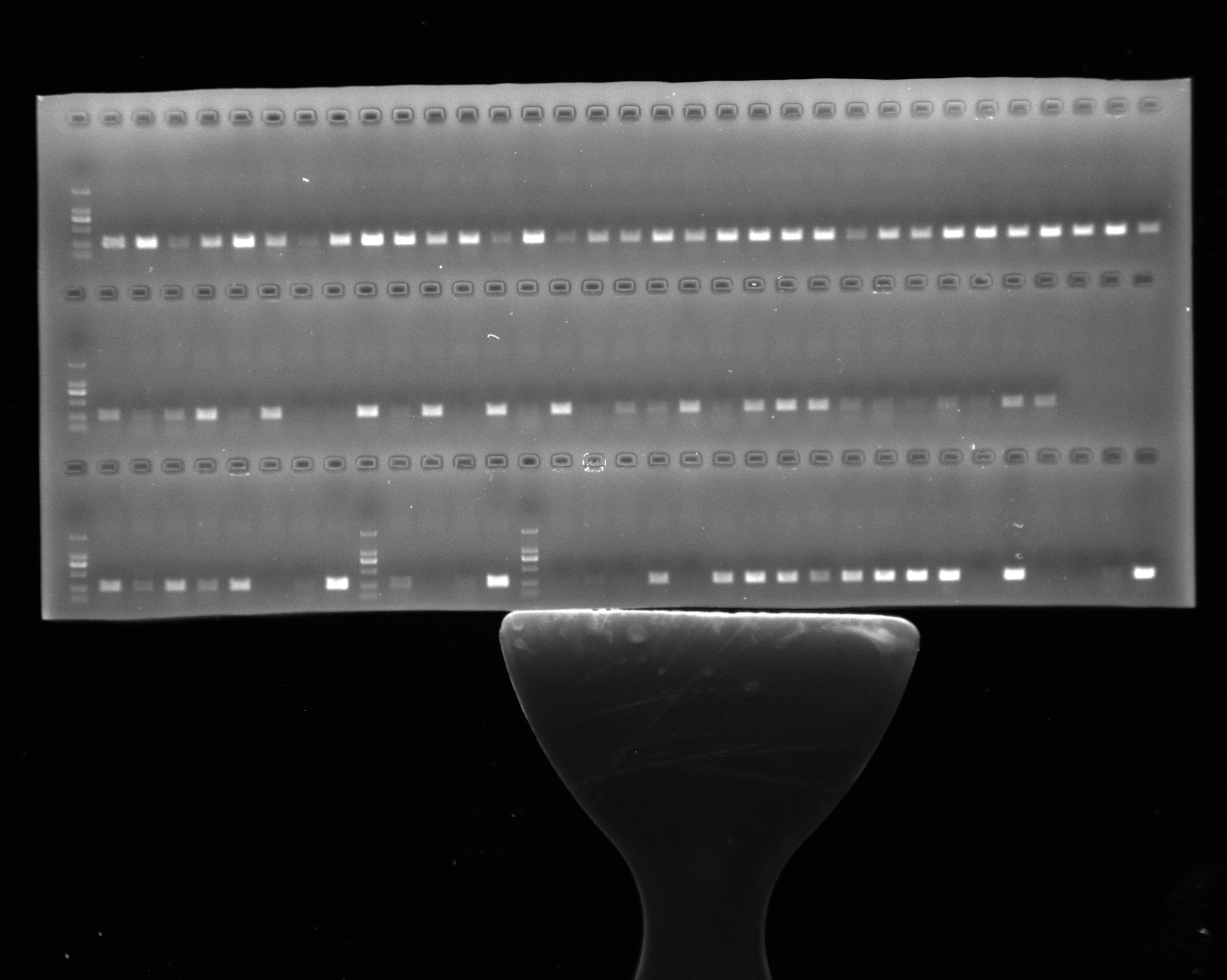
**

**Fig5a and Fig. S8**

**
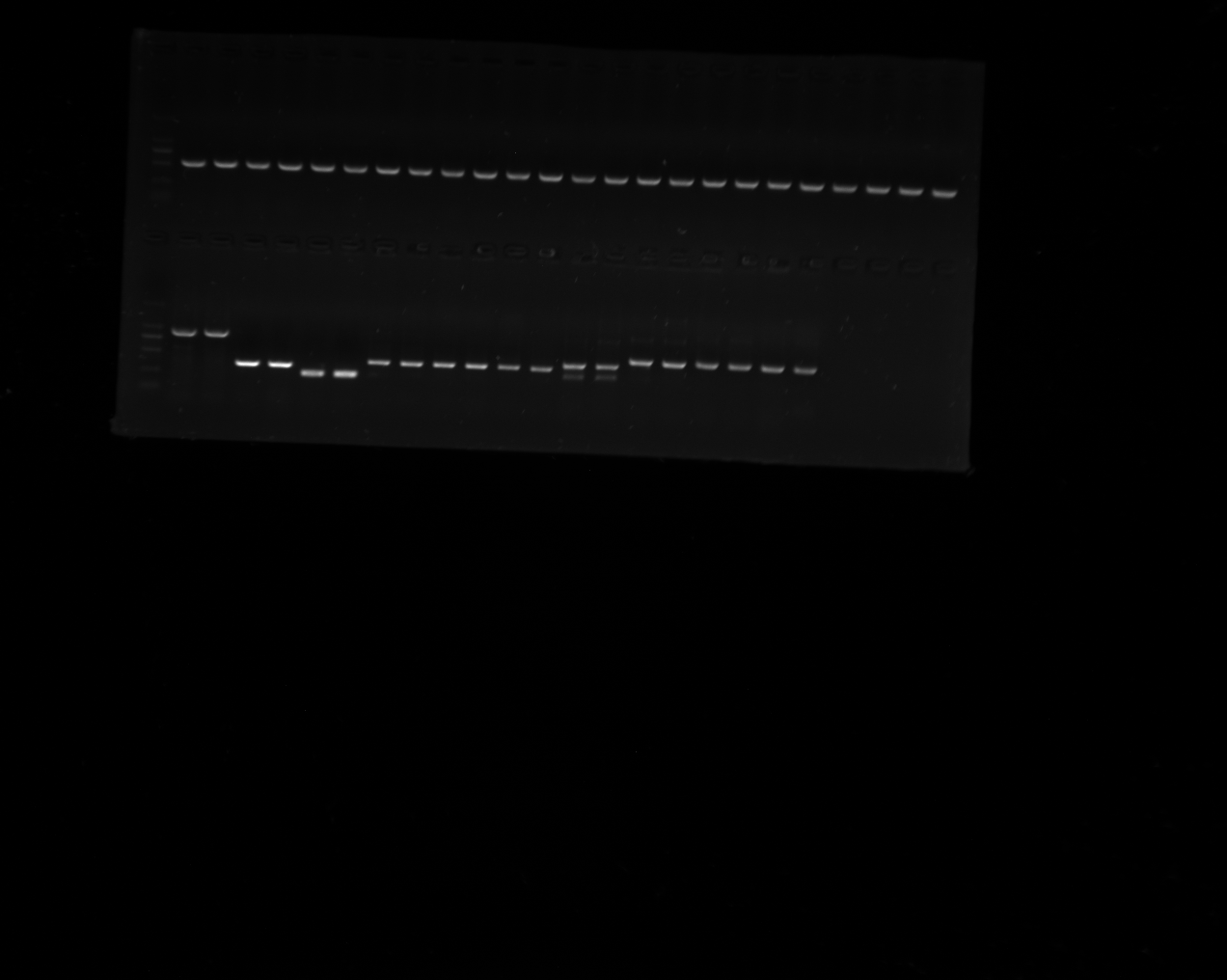
**

**Fig. S3**

Supplement: Supplementary file 16 — Additional file 16. [file 12864_2022_8549_MOESM16_ESM.docx]
