# Supplementary material for: Gene silencing, knockout and over-expression of a transcription factor ABORTED MICROSPORES (SlAMS) strongly affects pollen viability in tomato (Solanum lycopersicum)
Source: BMC Genomics. 2022 May 5;23(Suppl 1):346. doi: 10.1186/s12864-022-08549-x (PMC9069838; doi:10.1186/s12864-022-08549-x)
Supplement: Supplementary file 4 — Additional file 4: Fig. S4. VIGS-mediated silencing of SlAMS in tomato plants. a Infected with A. tumefaciens carrying pTRV2-SlAMS + pTRV1. b Infected with A. tumefaciens carrying pTRV2-PDS + pTRV1. c Negatively infected with A. tumefaciens carrying the basic vector pTRV2 + pTRV1. d Uninfected wild type. [file 12864_2022_8549_MOESM4_ESM.docx]

**
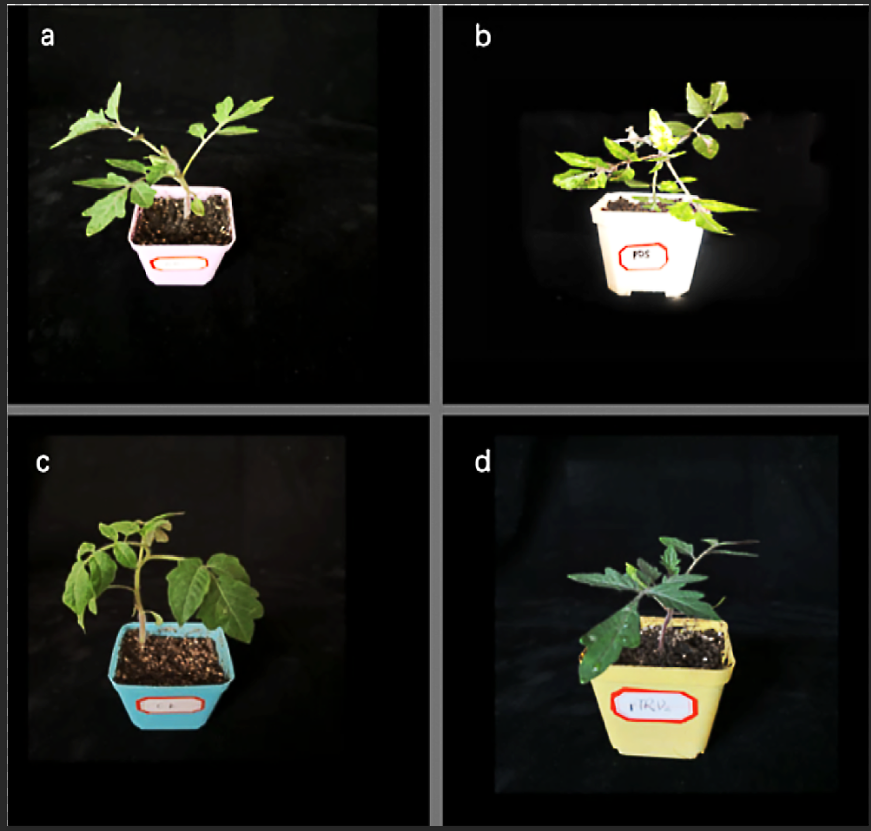
**

**Fig. S4** VIGS-mediated silencing of SlAMS in tomato plants. (a) Infected with A. tumefaciens carrying pTRV2-SlAMS+pTRV1. (b) Infected with A. tumefaciens carrying pTRV2-PDS+pTRV1. (c) Negatively infected with A. tumefaciens carrying the basic vector pTRV2+pTRV1. (d) Uninfected wild type.
