# Supplementary material for: Gene silencing, knockout and over-expression of a transcription factor ABORTED MICROSPORES (SlAMS) strongly affects pollen viability in tomato (Solanum lycopersicum)
Source: BMC Genomics. 2022 May 5;23(Suppl 1):346. doi: 10.1186/s12864-022-08549-x (PMC9069838; doi:10.1186/s12864-022-08549-x)
Supplement: Supplementary file 5 — Additional file 5: Fig. S5. The performance of inflorescence of SlAMS-silenced, −knockouted and -overexpressed tomato plants. a Untransformed wild type. b SlAMS-silenced plant. c SlAMS-knockouted plant. d SlAMS-overexpressed plant. [file 12864_2022_8549_MOESM5_ESM.docx]

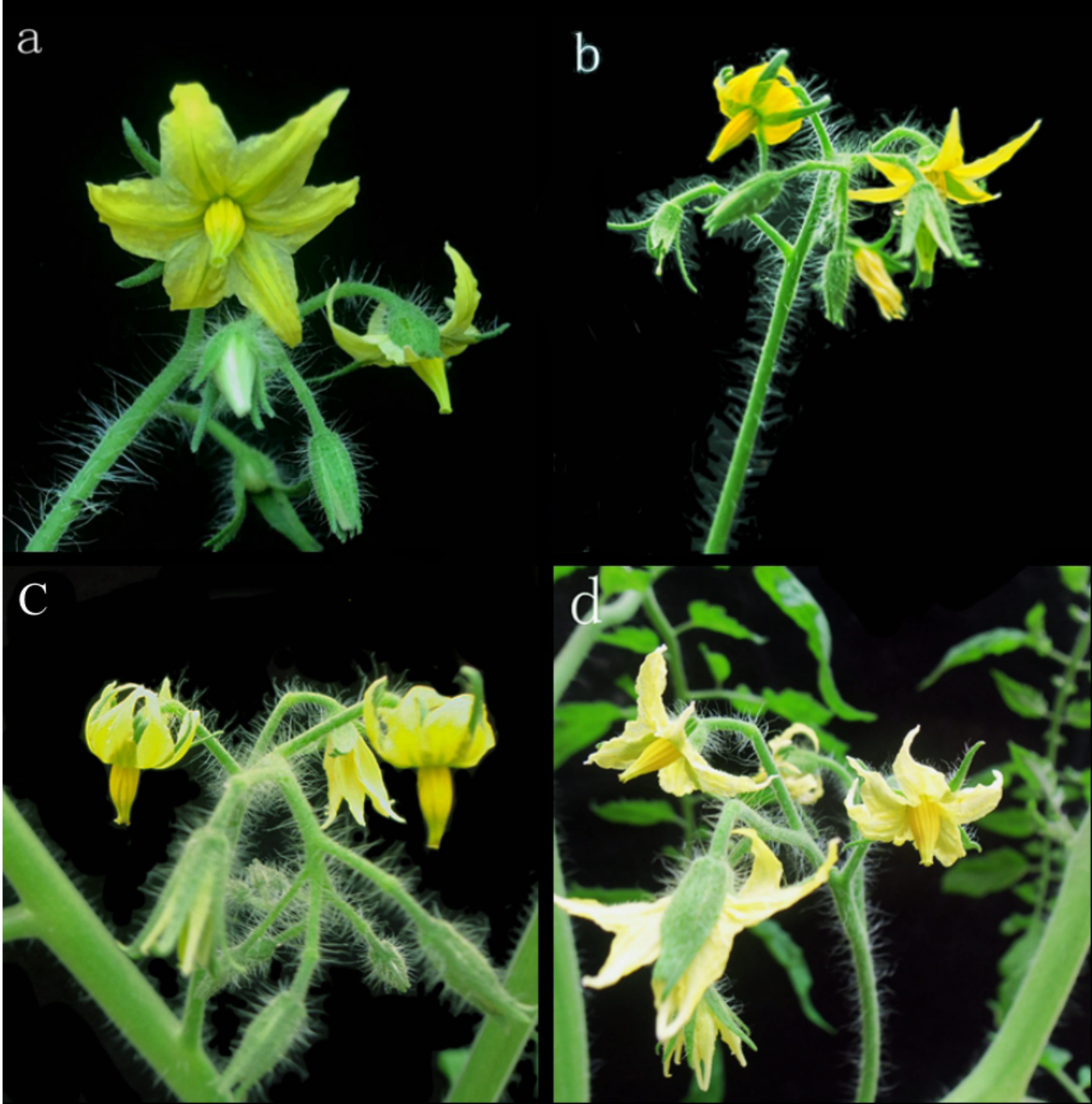


**Fig. S5** The performance of inflorescence of *SlAMS*-silenced, -knockouted and -overexpressed tomato plants. (a) Untransformed wild type. (b) *SlAMS*-silenced plant. (c) *SlAMS* -knockouted. (d) *SlAMS*-overexpressed plant.
