# Supplementary material for: Gene silencing, knockout and over-expression of a transcription factor ABORTED MICROSPORES (SlAMS) strongly affects pollen viability in tomato (Solanum lycopersicum)
Source: BMC Genomics. 2022 May 5;23(Suppl 1):346. doi: 10.1186/s12864-022-08549-x (PMC9069838; doi:10.1186/s12864-022-08549-x)
Supplement: Supplementary file 6 — Additional file 6: Fig. S6. PCR identification of the overexpression vector pCAMBIA2301-SlAMS. Lane A: 5 k Marker; Lane 1: -CK; Line2: SlAMS gene amplification products. [file 12864_2022_8549_MOESM6_ESM.docx]

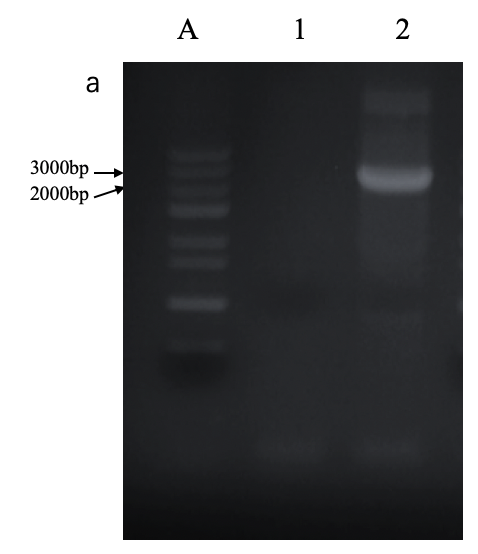


**Fig. S6** PCR identification of the overexpression vector pCAMBIA2301-SlAMS. Lane A: 5k Marker; Lane 1: -CK; Line2: SlAMS gene amplification products.
