# Supplementary material for: Gene silencing, knockout and over-expression of a transcription factor ABORTED MICROSPORES (SlAMS) strongly affects pollen viability in tomato (Solanum lycopersicum)
Source: BMC Genomics. 2022 May 5;23(Suppl 1):346. doi: 10.1186/s12864-022-08549-x (PMC9069838; doi:10.1186/s12864-022-08549-x)
Supplement: Supplementary file 7 — Additional file 7: Fig. S7. The Agrobacterium-mediated transformation of pCAMBIA230-SlAMS vector using tomato cotyledons. a pre-culture of cotyledons; (b) selection and regenerated culture; (c) seedling culture producing cotyledons; (d) transgenic plants transplanting after rooting culture. [file 12864_2022_8549_MOESM7_ESM.docx]

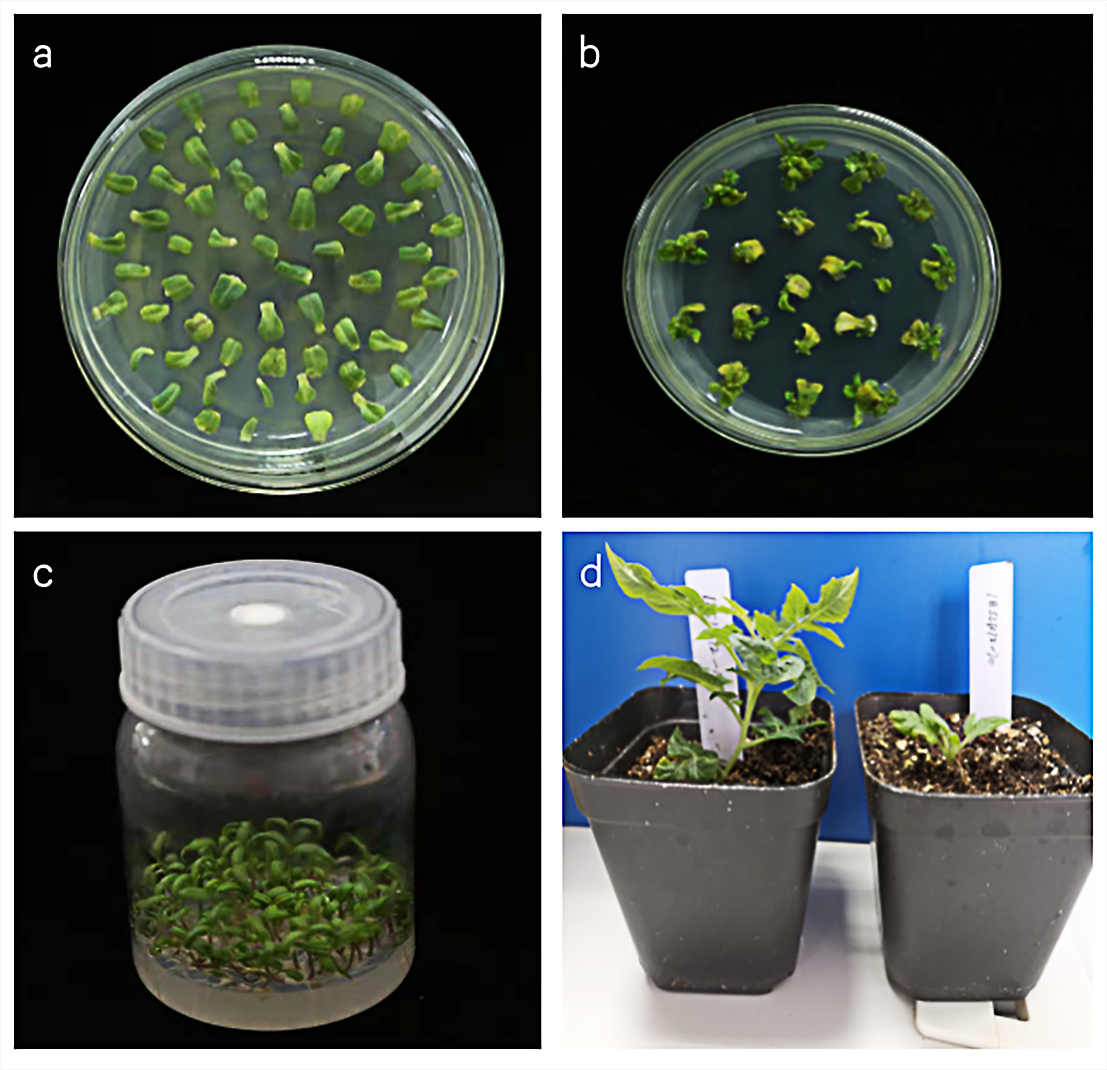


**Fig. S7** The *Agrobacterium-mediated* transformation of pCAMBIA230-*SlAMS* vector using tomato cotyledons. (a) pre-culture of cotyledons; (b)selection and regenerated culture; (c) seedling culture producing cotyledons; (d) transgenic plants transplanting after rooting culture.
