# Supplementary material for: Gene silencing, knockout and over-expression of a transcription factor ABORTED MICROSPORES (SlAMS) strongly affects pollen viability in tomato (Solanum lycopersicum)
Source: BMC Genomics. 2022 May 5;23(Suppl 1):346. doi: 10.1186/s12864-022-08549-x (PMC9069838; doi:10.1186/s12864-022-08549-x)
Supplement: Supplementary file 8 — Additional file 8: Fig. S8. PCR identification of tomato plants positively transformed with the pCAMBIA2301-SlAMS vector. Lanes 1–31: PCR products of Npt II; B: no target DNA; Lane N: nontransgenic plant; Lane P: pCAMBIA2301-SlAMS plasmid. [file 12864_2022_8549_MOESM8_ESM.docx]

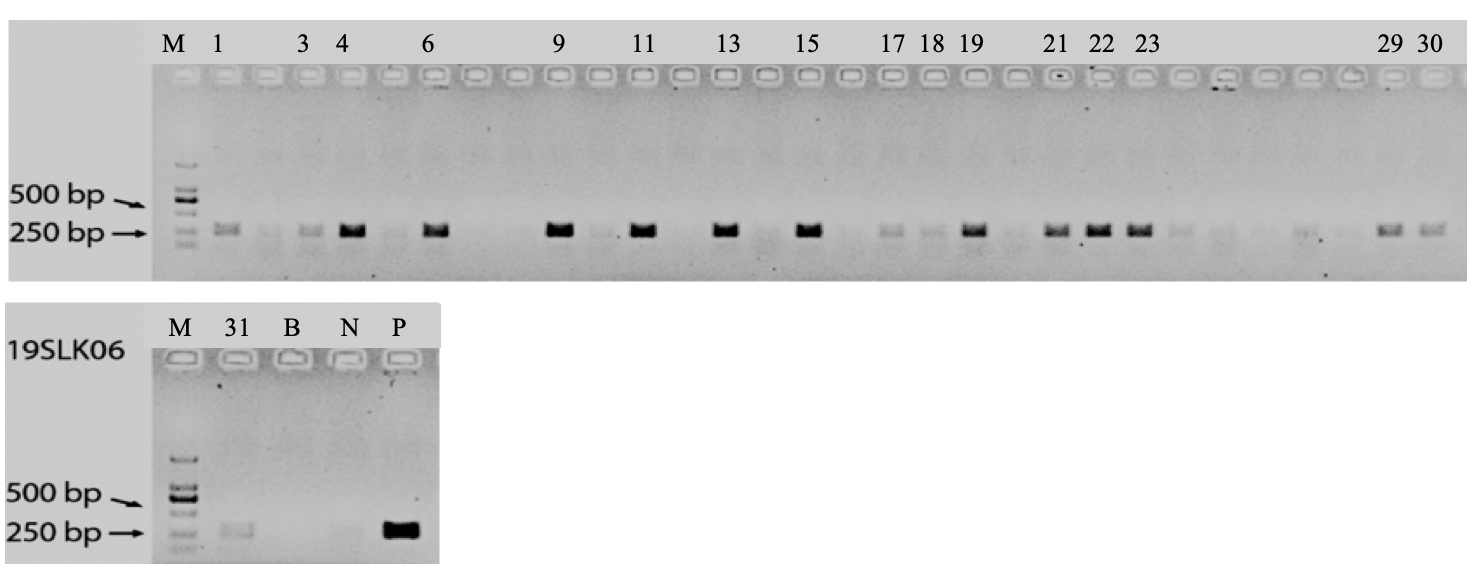


**Fig. S8** PCR Identification of tomato plants positively transformed with the pCAMBIA2301-*SlAMS* vector. Lanes 1–31: PCR products of *Npt* II; B: no target DNA; Lane N: nontransgenic plant; Lane P: pCAMBIA2301-*SlAMS* plasmid.
