# Supplementary material for: Gene silencing, knockout and over-expression of a transcription factor ABORTED MICROSPORES (SlAMS) strongly affects pollen viability in tomato (Solanum lycopersicum)
Source: BMC Genomics. 2022 May 5;23(Suppl 1):346. doi: 10.1186/s12864-022-08549-x (PMC9069838; doi:10.1186/s12864-022-08549-x)
Supplement: Supplementary file 9 — Additional file 9: Fig. S9. The map of pTRV2 vector used for the virus induced gene silencing (VIGS) of the tomato SlAMS gene. [file 12864_2022_8549_MOESM9_ESM.docx]

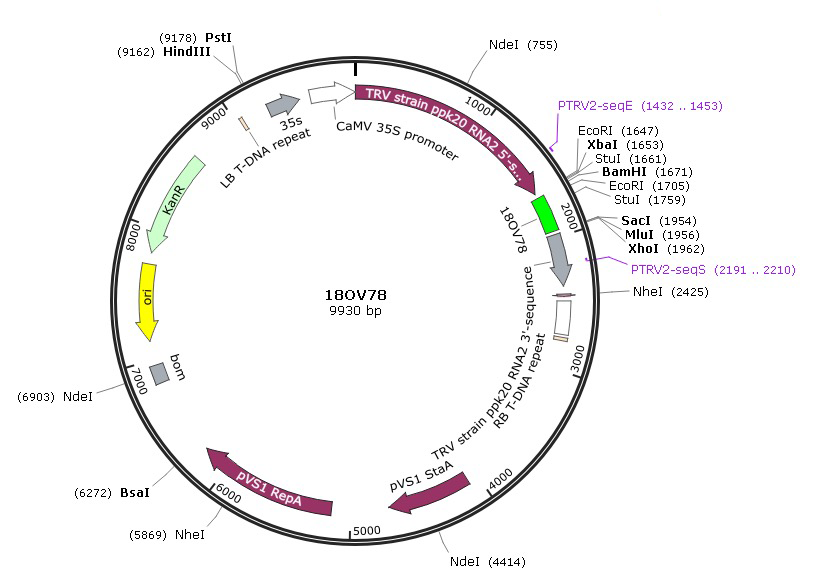


**Fig.S9** The map of pTRV2 vector used for the virus induced gene silencing (VIGS) of the tomato *SlAMS* gene.
