# Supplementary material for: Gene silencing, knockout and over-expression of a transcription factor ABORTED MICROSPORES (SlAMS) strongly affects pollen viability in tomato (Solanum lycopersicum)
Source: BMC Genomics. 2022 May 5;23(Suppl 1):346. doi: 10.1186/s12864-022-08549-x (PMC9069838; doi:10.1186/s12864-022-08549-x)
Supplement: Supplementary file 12 — Additional file 12: Table S1. The AMS gene sequences in Genebank used for nucleotide similarity analysis compared with the cloned SlAMS gene from tomato. [file 12864_2022_8549_MOESM12_ESM.docx]

**Table.S1** The *AMS* gene sequences in Genebank used for nucleotide similarity analysis compared with the cloned *SlAMS* gene from tomato.

| Gene name | Type | Origin of gene | Score (Bits) | E Value | Max Identity | Accession |
| --- | --- | --- | --- | --- | --- | --- |
| ABORTED MICROSPORES | transcript variant X1,mRNA | *Solanum lycopersicum* | 3315 | 0.0 | 100% | XM_019215077.2 |
| ABORTED MICROSPORES | transcript variant X1,mRNA | *Solanum pennellii* | 3114 | 0.0 | 98% | XM_027919037.1 |
| ABORTED MICROSPORES | transcript variant X1,mRNA | *Solanum tuberosum* | 2920 | 0.0 | 96% | XM_015310543.1 |
| AMS1 | complete cds,mRNA | *Capsicum annuum* | 2178 | 0.0 | 89% | MH230199.1 |
| ABORTED MICROSPORES | transcript variant X1,mRNA | *Capsicum annuum* | 2163 | 0.0 | 89% | XM_016682091.1 |
| ABORTED MICROSPORES | transcript variant X4,mRNA | *Solanum tuberosum* | 2095 | 0.0 | 95% | XM_015310545.1 |
| ABORTED MICROSPORES | complete cds,mRNA | *Physalis pubescens* | 2001 | 0.0 | 87% | MH319844.1 |
| ABORTED MICROSPORES-like | mRNA | *Nicotiana tabacum* | 1168 | 0.0 | 85% | XM_016592743.1 |
| ABORTED MICROSPORES | transcript variant X1,mRNA | *Nicotiana tomentosiformis* | 1162 | 0.0 | 85% | XM_009618243.3 |
| ABORTED MICROSPORES-like | transcript variant X1,mRNA | *Nicotiana tabacum* | 1140 | 0.0 | 87% | XM_016659971.1 |
| ABORTED MICROSPORES | transcript variant X1,mRNA | *Nicotiana sylvestris* | 1134 | 0.0 | 86% | XM_009787837.1 |
| ABORTED MICROSPORES-like | mRNA | *Coffea arabica* | 187 | 0.0 | 78% | XM_027268300.1 |
