# Supplementary material for: Gene silencing, knockout and over-expression of a transcription factor ABORTED MICROSPORES (SlAMS) strongly affects pollen viability in tomato (Solanum lycopersicum)
Source: BMC Genomics. 2022 May 5;23(Suppl 1):346. doi: 10.1186/s12864-022-08549-x (PMC9069838; doi:10.1186/s12864-022-08549-x)
Supplement: Supplementary file 13 — Additional file 13: Table S2. Primer sequences used in this paper (designed using Primer 5.0). [file 12864_2022_8549_MOESM13_ESM.docx]

**Table S2.** Primer sequences used in this paper (designed using Primer 5.0).

| Primer | Sequences (5′-3′) |
| --- | --- |
| AMSad_1_ | CAAATGAGTTAAACCTAGCTATC |
| AMSad_2_ | GAAAAA GCAGAAGTACATTATTAC |
| AMS-F1 | GAAGGCCTCCATGGGGATCCCAGATGACGACGGTACTGGT |
| AMS-R1 | GCCTCGAGACGCGTGAGCTCGTTCACGAAACACCTTCACAA |
| AMS-F2 | ATGTATGATGCAGGTTTCTTGA |
| AMS-R2 | ATGTATGATGCAGGTTTCTTGA |
| qAMS-F | TGGATTTGTTGCTGCTGTGG |
| qAMS-R | TGTCTCTTTCTCCTTCTGTTGGG |
| Actin-F | CTTCCCTCAGCACCTTCCAG |
| Actin-R | TGGTCCAGTAGGAAATAAGAAGTC |
| pTRV2-seqE | CACATATTCGCACGTATGAAGT |
| pCRISPR-Sens | TTGGCCTTCTCTGGCGCTAGA |
| pCRISPR-Anti | AACTCTAGCGCCAGAGAAGGC |
| pCRISPR-SeqE | GATGAAGTGGACGGAAGGAAGGAG |
| NPTIIF68 | ACTGGGCACAACAGACAATCG |
| NPTIIR356 | GCATCAGCCATGATGGATACTTT |
| 2301-AMSF | CGGATCCATGGAACTCATGCATCT |
| 2301-AMSR | TTAGTGATGGTGGTGTGTATGGA |
