# Supplementary material for: Gene silencing, knockout and over-expression of a transcription factor ABORTED MICROSPORES (SlAMS) strongly affects pollen viability in tomato (Solanum lycopersicum)
Source: BMC Genomics. 2022 May 5;23(Suppl 1):346. doi: 10.1186/s12864-022-08549-x (PMC9069838; doi:10.1186/s12864-022-08549-x)
Supplement: Supplementary file 14 — Additional file 14: Table S3. Accession numbers of the AMS proteins used in the phylogenetic tree. [file 12864_2022_8549_MOESM14_ESM.docx]

**Table S3**. Accession numbers of the AMS proteins used in the phylogenetic tree.

| Species Name | Accession Number |
| --- | --- |
| *Abrus precatorius* | XP_027364813.1 |
| *Arabidopsis thaliana* | NP_179283.2 |
| *Arachis hypogaea* | XP_025674351.1 |
| *Brassica napus* | XP_022562176.1 |
| *Brassica oleracea var.oleracea* | XP_013598295.1 |
| *Camelina sativa* | XP_010489298.1 |
| *Capsicum annuum* | XP_016537577.1 |
| *Cucumis melo var. makuwa* | KAA0042490.1 |
| *Cucumis melo* | XP_016902324.1 |
| *Cynara cardunculus var.scolymus* | XP_024988957.1 |
| *Glycine max* | XP_025979976.1 |
| *Glycine soja* | RZB89530.1 |
| *Helianthus annuus* | XP_022028120.1 |
| *Lactuca sativa* | XP_023751248.1 |
| *Momordica charantia* | XP_022135617.1 |
| *Nicotiana attenuata* | OIT32810.1 |
| *Physalis pubescens* | AZB50351.1 |
| *Raphanus sativus* | XP_018437312.1 |
| *Solanum lycopersicum* | QDO73362.1 |
| *Solanum pennellii* | XP_027774838.1 |
| *Solanum tuberosum* | XP_006351593.1 |
| *Tagetes erecta* | AVX48617.1 |
| *Vigna angularis* | XP_017413235.1 |
| *Vigna radiata var.radiata* | XP_022640713.1 |
| *Vigna unguiculata* | XP_027936415.1 |
