# Supplementary material for: Gene silencing, knockout and over-expression of a transcription factor ABORTED MICROSPORES (SlAMS) strongly affects pollen viability in tomato (Solanum lycopersicum)
Source: BMC Genomics. 2022 May 5;23(Suppl 1):346. doi: 10.1186/s12864-022-08549-x (PMC9069838; doi:10.1186/s12864-022-08549-x)
Supplement: Supplementary file 15 — Additional file 15: Table S4. The web sites used for bioinformatics analyses. [file 12864_2022_8549_MOESM15_ESM.docx]

**Table S4.** The web sites used for bioinformatics analyses.

| Name of software | Website |
| --- | --- |
| BLAST | <http://www.ncbi.nlm.gov/blast> |
| DNAMAN | <http://www.lynnon.com/> |
| MEGAX | <http://www.megasoftware.net/> |
| PredictProte | [http://www.](http://www.cbi.pku.edu.)predictprotein.org/home |
| PSORT Π predict | <http://psort.hgc.jp/form2.html> |
| SOPMA | <http://npsa-pbil.ibcp.fr/> |
| SWISS-MODEL | <http://swissmodel.expasy.org/> |
